# Willingness to engage in marine conservation through eDNA-informed citizen science on whale-watching platforms

**DOI:** 10.1038/s41598-025-26209-4

**Published:** 2025-11-25

**Authors:** Eleonora Barbaccia, Lauren Kelly Rodriguez, Belén García Ovide, Mario Gabualdi, Enrico Villa, Maddalena Jahoda, Caterina Lanfredi, Marianne Helene Rasmussen, Michael Traugott, Bettina Thalinger, Arianna Azzellino

**Affiliations:** 1 Civil and Environmental Engineering Department (DICA), Politecnico di Milano, Piazza Leonardo da Vinci, 32, Milan, 20133 MI Italy; 2 Applied Animal Ecology Research Unit, Department of Zoology, University of Innsbruck, Innsbruck, 6020 Austria; 3 The University of Iceland´s Research Center in Húsavík, Hafnarstétt 3, Húsavík, 640 Iceland; 4 Cetacean Watching Lda, Cais da Madalena, Madalena do Pico, Azores, 9950-305 Portugal; 5 Tethys Research Institute, c/o Acquario Civico, via G.B. Gadio, 2, Milan, 20121 Italy

**Keywords:** Willingness to pay (WTP), Questionnaire survey, Environmental DNA (eDNA), Citizen science, Public engagement, Environmental education, Biodiversity monitoring, Ecology, Ecology, Environmental social sciences, Environmental studies

## Abstract

Marine biodiversity faces challenges due to climate change, pollution, and human activities. Conservation efforts are often constrained by limited resources and public disengagement. This study evaluates the potential benefits of incorporating environmental DNA (eDNA) sampling and citizen science into whale-watching tours to raise environmental awareness and promote marine conservation. A Likert-scale questionnaire evaluating attitudes, knowledge, and willingness to contribute to conservation through financial support, behavioral changes, and volunteering was administered to 224 participants across the Pelagos Sanctuary (Italy), the Azores (Portugal), and Skjálfandi Bay (Iceland). Environmental awareness, interest in environmental DNA (eDNA), and higher education levels were identified as significant predictors of willingness to pay (WTP). Conversely, higher ticket prices negatively impacted financial commitment. The majority of respondents reported an increased awareness of marine biodiversity and eDNA, along with a strong sense of personal responsibility toward marine protection. The study outlined various participant profiles, indicating how age, education, and ecotourism experience influence conservation attitudes. Specifically, younger individuals who are highly educated and have prior whale-watching experience were more inclined to provide financial support and engage in citizen science activities. These findings demonstrate that integrating citizen science-based activities and environmental DNA (eDNA) sampling, into whale-watching tours is an effective way to foster meaningful pro-environmental engagement. This model presents a scalable and cost-effective approach to enhancing public participation in marine conservation and biodiversity monitoring, with potential applications extending to broader marine governance and ecotourism settings.

**Supplementary Information:**

The online version contains supplementary material available at 10.1038/s41598-025-26209-4.

## Introduction

Marine ecosystems are increasingly threatened by climate change^1^, pollution^2^, and anthropogenic pressures^3^, demanding for innovative approaches to biodiversity conservation. Monitoring and protecting marine biodiversity remain particularly challenging due to the vast spatial scales involved, logistical limitations, and persistent constraints in funding availability^4–6^. Although international frameworks and marine protected areas (MPAs) have been established, conservation efforts are frequently hampered by inadequate financial resources^7^, undermining the capacity to track biodiversity trends systematically^8,9^. This issue is especially concerning in the context of accelerating climate change, as rising ocean temperatures, acidification, and ecological shifts pose substantial threats to species survival and ecosystem stability^10^. Without sustained and widespread biodiversity monitoring, many species declines may remain undetected, limiting the effectiveness of conservation responses^11,12^.

The success of conservation efforts is strongly related to how people perceive marine biodiversity. In this context, public engagement is increasingly recognized as a vital complement to conventional conservation strategies^13,14^. Citizen science initiatives that engage non-experts in environmental monitoring offer the dual benefit of generating valuable biodiversity data while simultaneously bringing the public closer to applied conservation research and empowering them to play an active role in biodiversity protection^15–21^. Moreover, numerous studies have demonstrated that immersive nature-based tourism experiences can positively shape pro-conservation attitudes, often translating into a greater willingness to contribute financially to environmental protection efforts^22–25^. For instance, Petcharat et al^26^. found that residents expressed a clear willingness to pay for the sustainable development of protected areas, indicating that direct engagement with natural resources fosters pro-conservation attitudes. Sayan et al^27^. conducted a pilot study on popular Mediterranean beaches in Antalya, Turkey, and found that tourists were willing to financially support beach conservation efforts, particularly to protect sea turtle nesting areas, when made aware of the ecological value of the site.

Whale-watching, a rapidly expanding sector of marine ecotourism, offers an ideal platform for such initiatives by enabling participants to directly observe marine biodiversity while contributing to research and conservation goals^28,29^. For instance, La Manna et al^30^. demonstrated that dolphin watching tours in Sardinia and Lošinj, Croatia, significantly increased participants’ environmental awareness and sense of responsibility for marine conservation. Visitors reported an enhancement in their attitudes toward conservation following the tours, indicating that immersive marine ecotourism experiences can effectively cultivate pro-environmental behaviors, particularly when coupled with education and responsible management. Similar positive outcomes were observed by Ioppolo et al. (2013) in a sustainable marine ecotourism program along the Sardinian coast, where participatory coastal management strategies contributed to increased environmental engagement among both tourists and local stakeholders (Ioppolo et al. 2013).

Willingness to pay (WTP) is a key indicator of public support in conservation efforts, particularly when linked to active participation in citizen science initiatives. Research shows that financial contributions to conservation are influenced by environmental awareness, personal connection to nature, income level, and the perceived urgency of ecological threats^31–35^. For instance, Lew^36^found that visitors to marine protected areas (MPAs) were more likely to pay conservation fees when objectives and benefits of their contributions were clearly communicated. In the context of marine megafauna, Malinauskaite et al^37^. estimated that Icelandic residents were willing to pay approximately €45 per year to expand the Faxaflói Bay whale sanctuary, demonstrating the economic value attributed to the protection of iconic marine species. More recently, Gelcich et al^38^. reported that Chilean coastal communities, including tourists, demonstrated strong WTP for participatory monitoring schemes involving marine biodiversity, highlighting the role of citizen involvement in sustaining local marine governance. These findings suggest that incorporating educational and participatory elements into marine ecotourism can enhance both awareness and economic support for biodiversity protection. While Barbaccia et al^15^. investigated how the combination of eDNA sampling and citizen science within whale-watching tours influenced participants, the present study explicitly willingness to pay (WTP) for biodiversity monitoring in this context. Conducted within the Biodiversa + eWHALE project, explores this gap through a cross-national survey in the *Pelagos* Sanctuary (Italy), Azores (Portugal), and Skjálfandi Bay (Iceland). The study assesses participants’ willingness to pay (WTP), willingness to adjust behaviors (WTAB), and willingness to volunteer (WTV), thereby offering novel insights into the potential of integrating eDNA biodiversity monitoring with whale-watching-based citizen science as a scalable conservation model.

## Materials and methods

### Study area

**Fig. 1:**
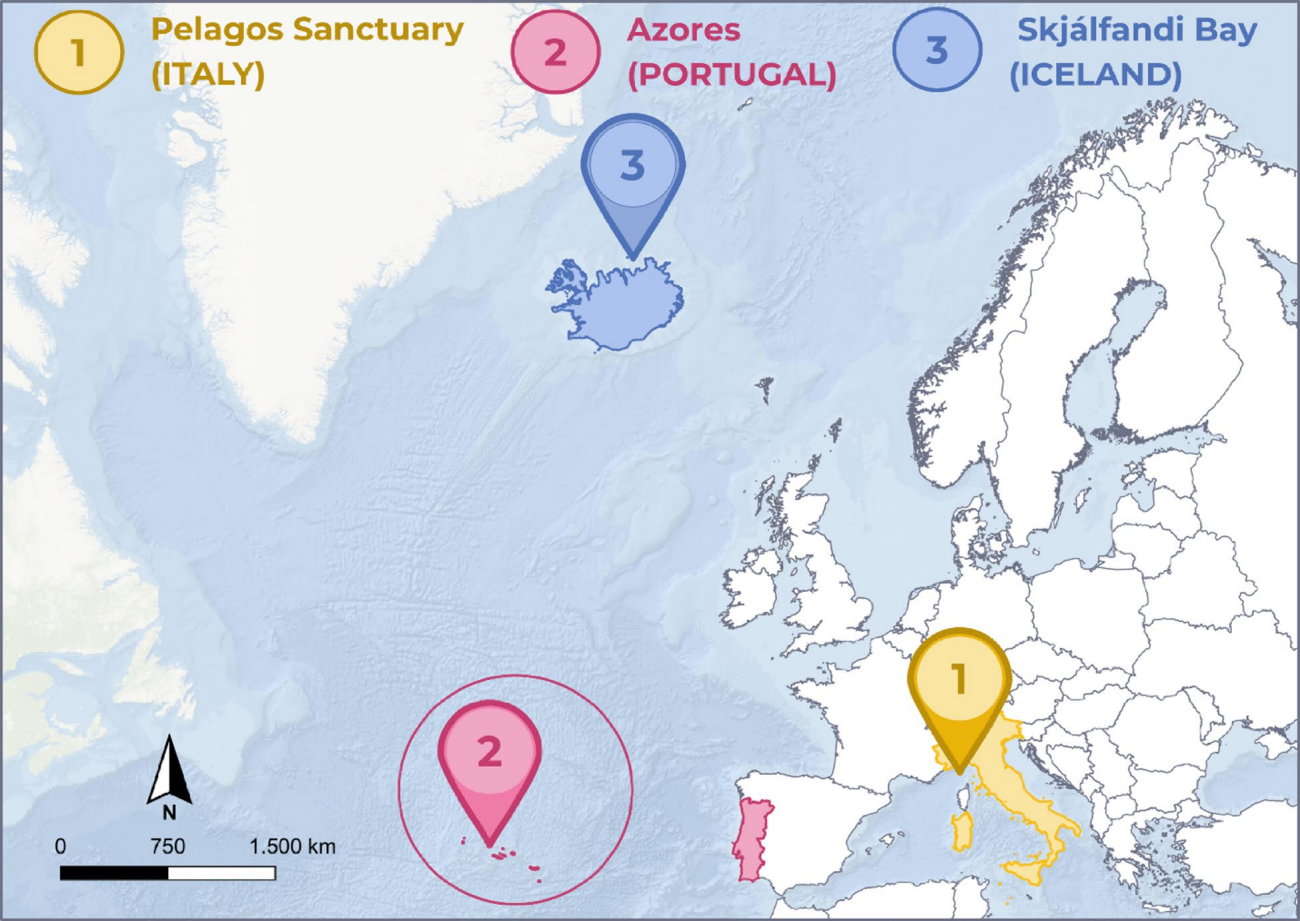
Geographical distribution of survey sites across the Pelagos Sanctuary (Italy), the Azores (Portugal), and Skjálfandi Bay (Iceland). The map was created in QGIS 3.28 (https://qgis.org*)* using Esri basemap services; CRS: WGS 84. The layout and labels were finalized in Canva (https://www.canva.com*).*

A questionnaire survey was carried out in the Pelagos Sanctuary (Italy), the Azores (Portugal), and Skjálfandi Bay (Iceland) as part of the eWHALE project, a BioDiversa + initiative focused on education and marine conservation through citizen science and environmental DNA sampling (Fig. 1).

These study areas were selected due to their ecological diversity, namely the presence of different cetacean species, and their established whale-watching industries, thus providing a range of contexts for the aims of this research.

In Italy, whale-watching is conducted along the Ligurian coast within the Pelagos Sanctuary, a transboundary marine protected area in the northwestern Mediterranean Sea. This Sanctuary, established by Italy, France, and Monaco in 2002, spans approximately 87,500 km² and is home to eight resident species of marine mammals (Pelagos Sanctuary, 1999). Its design was based on oceanographic features such as the Ligurian Front, which supports high zooplankton concentrations, forming vital feeding grounds for species like fin whales and striped dolphins^39^. Despite its ecological significance, the Sanctuary faces major conservation challenges. Heavy maritime traffic, particularly near ports like Genoa, contributes to underwater noise pollution and increases the risk of ship strikes, especially for species such as sperm whales and fin whales^40,41^. Moreover, plastic pollution and chemical contaminants are critical threats to marine life in the Sanctuary. Fin whales are particularly at risk from microplastic ingestion due to pollution from rivers, coastal runoff, and shipping lanes^42^. Toxicological stress linked to pollutants such as PCBs and DDTs has been found in stranded bottlenose dolphins, compromising immune health and increasing susceptibility to disease^43^. These findings are echoed by studies showing broader declines in species diversity in more disturbed zones, such as near Genoa, compared to less trafficked areas like Imperia^44^. To counteract these issues, several initiatives aim to improve conservation management. These include spatial planning to identify high-risk zones^45^, efforts to regulate vessel speeds (Frassà et al^46^.), and the expansion of the Natura 2000 network to better encompass offshore areas crucial to cetacean habitats^47^. Public education campaigns and responsible whale-watching guidelines are also being implemented to increase awareness and reduce human impact^48^.

Iceland has emerged as a premier destination for marine biodiversity, especially known for hosting several cetacean species such as blue whales, minke whales, humpback whales, and killer whales^49^. The cold, nutrient-rich waters of the North Atlantic, particularly around Faxaflói and Skjálfandi Bays, provide abundant food resources during the summer, creating ideal feeding grounds^50,51^. Whale-watching has become a significant economic and cultural feature in Iceland, especially in coastal communities like Húsavík, often referred to as the “whale capital” of the country. Once reliant on whaling, Húsavík has transitioned into a major hub for cetacean-based ecotourism, with whale-watching now a cornerstone of the local economy^52^. Studies have highlighted that whale-watching in Iceland generates millions in revenue annually, more than what was ever earned through commercial whaling^53^. Environmental sustainability has also become a focus, as Iceland has taken steps to protect vital marine areas, such as the Breiðafjörður Bay, home to diverse marine life and critical cetacean habitats. Moreover, areas like Skjálfandi Bay are included within whale sanctuaries to mitigate human impact^37^. Nevertheless, climate change and whale-watching activity are bringing new conservation challenges. Recent studies show that boat noise and warming waters may disrupt whale foraging and migration patterns^54,55^. The co-existence of whale-watching and traditional fishing also requires careful management, especially as some fishermen still view cetaceans as competitors for fish stocks^56^. Efforts to integrate sustainable boating practices, such as transitioning to electric whale-watching vessels, show promise in reducing the environmental footprint of tourism^57^.

The Azores, a remote mid-Atlantic archipelago, has become one of the world’s premier destinations for whale-watching due to its extraordinary marine biodiversity and favorable oceanographic conditions^58^. The surrounding deep, nutrient-rich waters, influenced by complex current systems like the North Atlantic Drift and local upwellings, create ideal feeding grounds for a wide variety of cetaceans. These include resident populations such as sperm whales and several dolphin species, as well as migratory giants like blue, fin, and humpback whales^59^. Historically a whaling hub, the Azores, has undergone a remarkable shift toward conservation-based ecotourism. Since the cessation of whaling in the 1980 s, whale-watching has grown into a major economic activity and cultural hallmark of the region, especially in islands like Pico and São Miguel^60^. The industry not only provides jobs and supports sustainable tourism but also raises public awareness about marine conservation and supports scientific research through citizen science initiatives and collaborations with local universities. To ensure the long-term protection of its marine resources, the Azores have established an extensive network of marine protected areas, including the Azores Marine Park (AMP). Created to unify and expand various offshore MPAs, the AMP spans over 110,000 km² and serves as the foundation of the archipelago’s marine conservation strategy^61^. It encompasses ecologically significant features such as seamounts, deep-sea coral gardens, hydrothermal vents, and bird foraging areas. These habitats are crucial for both endemic and migratory species, and the AMP’s comprehensive management framework aims to protect them from threats like overfishing, seabed mining, and unregulated tourism^62^. While the Azores Marine Park represents a strong commitment to ocean stewardship, its success depends on improving enforcement, stakeholder coordination, and scientific monitoring systems^63^.

### Whale-watching operators

This study involved three partners in the eWHALE project: a non-profit research organization, running a citizen-science driven project (Tethys Research Institute) and two commercial whale-watching companies (North Sailing & CW Azores), hereafter collectively referred to as “whale-watching operators” irrespective of their commercial or non-profit status or of whether their activities are research-oriented.

Each operator offers a unique experience while sharing a common commitment to marine conservation.

In Italy, the Tethys Research Institute, a research organization dedicated to the study and conservation of large marine vertebrates, has been engaging the public in scientific research since 1990, offering week-long citizen science expeditions in the Ligurian Sea, and providing a fully immersive and research-driven experience. Educational activities are integrated throughout the expedition and include daily lessons on cetacean biology, ecology, and research techniques. Participants are directly involved in scientific data collection, such as visual surveys, behavioral observations, and acoustic monitoring, gaining hands-on experience in cetacean research. This extended format promotes continuous interaction with researchers and a deep understanding of marine conservation challenges.

In Iceland, the main operator, North Sailing, based in Húsavík conducts multiple 3–3.5-hour tours per day in Skjálfandi Bay, with daily frequencies ranging from 1 to 6 daily in spring to 12–15 in summer. Information is primarily delivered through microphone commentary by a marine biologist during navigation, ensuring accessibility for all passengers. In addition, the company also offers a weekly 3.5-hour citizen science program (“Whale Sails and Science”) during the summer, as well as two annual week-long expeditions focused on microplastic monitoring in Northeast Iceland.

In the Azores, the operator CW Azores also conducts several three-hour excursions per day. Unlike the Icelandic model, each tour begins with a private pre-departure briefing delivered to small groups. During these sessions, staff introduce the main cetacean species that may be encountered and provide basic guidance on responsible wildlife observation. This face-to-face format allows for personalized interaction and prepares participants before embarking on the tour.

Despite differences in format, timing, and intensity, all three operators are united in their commitment to marine stewardship. Through a variety of educational approaches including citizen science engagement, live onboard narration, and targeted pre-tour briefings, they promote environmental awareness and foster public engagement in marine conservation. Their diverse approaches demonstrate how educational content can be effectively adapted to different operational contexts, from long-distance scientific cruises to high-volume commercial whale-watching.

### Survey design

A voluntary, anonymous survey (see Supplementary Material 1) was given to participants at the end of the whale-watching experience between spring and fall of 2024. The 10-item questionnaire combined multiple-choice questions and Likert-scale statements ranging from “Strongly Disagree” to “Strongly Agree.” It was designed to comprehensively assess participants’ knowledge, perceptions, and environmental awareness. Based on the results of a previous survey conducted in the summer of 2023^15^, the 2024 version expanded its focus to include the WTP of whale-watching participants for marine conservation.

Six demographic questions (Q1–Q6) were included to assess the composition of the participant sample. These covered the country where the whale-watching activity took place (Pelagos Sanctuary (Italy), Azores (Portugal), and Skjálfandi Bay (Iceland)), participants’ country of origin, age group, gender, education level, and professional background. Additionally, participants were asked to indicate the frequency of their whale-watching experiences (Q7), allowing for differentiation between first-time and repeat participants.

Question 8 explored the factors influencing participants’ choice of whale-watching operator. Specifically, respondents were asked to rate the importance of several selection criteria, such as the price of the ticket, which may influence accessibility, the operator’s affiliation with conservation initiatives, which could signal environmental responsibility, and customer reviews on platforms like TripAdvisor or Google, which are often used as trust indicators by prospective guests. Participants also evaluated the importance of ethical conduct, such as adhering to whale-watching guidelines (e.g., maintaining safe distances from whales). This probed the balance between immersive experiences and ethical wildlife interactions. Educational components were also evaluated, such as opportunities to learn about marine biodiversity and conservation programs like eWHALE, including how to get involved. Finally, participants were asked to consider the value of learning about environmental DNA (eDNA) and its role in biodiversity monitoring. This reflects the growing integration of citizen science and public engagement in marine research.

Attitudes toward marine conservation were assessed using nine Likert-scale statements (Q9), each designed to explore different aspects of participants’ environmental values and behavioral intentions. These statements covered a range of key themes, including emotional concern, perceived responsibility, and willingness to act. Participants were asked to express their level of agreement with statements such as: *“I am worried about the health of the marine environment”* and *“Plastic pollution in the oceans poses a significant threat to marine life”*, both reflecting general environmental concern. Broader ecosystem implications were addressed through the statement *“The loss of marine biodiversity can negatively affect humans”*, which emphasized the interconnectedness of environmental and human well-being. Scientific awareness was assessed with the item *“Environmental DNA (eDNA) is a revolutionary way to safely study marine life without the need for invasive or costly sampling efforts”*, gauging familiarity with innovative, non-invasive research methods. Personal responsibility and ethical engagement were explored through statements such as *“I feel a personal obligation to protect the marine environment”* and *“Cleaning products and plastics that I use on a daily basis can have a negative effect on whales*,* dolphins*,* and the marine environment”*. Finally, three items measured participants’ willingness to take concrete action: *“I am willing to contribute financially to support marine conservation efforts”*; *“I am willing to adjust my behavior if it is necessary to protect the marine environment”*; and *“I am willing to actively participate by volunteering to support marine conservation*,* such as data collection.”* These statements provided insight into the depth of participants’ commitment to conservation beyond passive support.

### Statistical analysis

The survey responses were analyzed using IBM SPSS Statistics software (version 29; IBM Corp., 2020). Descriptive statistics and chi-square tests were employed to analyze demographic, behavioral, and attitudinal data in order to identify patterns and relationships. Due to the low sample size, which sometimes violated asymptotic assumptions, we confirmed the chi-square test’s asymptotic p-value with a Monte Carlo Randomization Analysis based on 10,000 samples, providing a 99% confidence interval for the p-value estimate. To explore the factors influencing individuals’ WTP for marine conservation, a Factor Analysis (hereinafter FA) was conducted to uncover the underlying dimensions of respondents’ attitudes, knowledge, and behaviors related to conservation. FA was obtained through a preliminary Principal Components Analysis which extracted the eigenvalues and eigenvectors from the covariance matrix of the original variances. FA was used to lessen the impact of less significant parameters within each component. This was done by rotating the PCA axes using the Varimax rotation criterion, maintaining orthogonality. Factors were chosen based on the “eigenvalue higher than 1” criterion, discarding those explaining less variance than an original variable. This method selected a few factors to describe the dataset with minimal information loss.

The factor analysis results guided a Hierarchical Cluster Analysis (hereinafter HCA) based on a Euclidean Distance matrix, and using Ward’s as Cluster method^64^. HCA was used to categorize respondents by shared motivations and behaviors. Finally, a stepwise logistic regression analysis was used to assess the best predictors for WTP for conservation efforts. For Logistic Regression, WTP was recorded into two classes: Agree (in favor) and Disagree/Neutral (not in favor). The Odds Ratio (OR) was utilized to assess the strength and correlation of the predictors in relation to the outcome so OR reveals the ratio of the odds of being in favor of WTP compared to the odds of not being in favor.

## Results

A total of 224 survey responses were collected: 67 (29.9%) from Italy, 37 (16.5%) from Iceland, and 120 (53.6%) from the Azores.

 Although 224 questionnaires were collected in total, only 168 complete cases were available for the demographic and factor analyses. The reduction in sample size is due to listwise exclusion of respondents with missing values in the relevant variables, including the exclusion of the small non-binary gender category (*n*=1; 0.4%), which could not be analyzed separately due to limited sample size. For chi-square tests, the effective N varied slightly (e.g., 223, 222, 221) depending on the number of missing responses for each item. Participants’ characteristics (gender, age class, and level of education) are shown in Table S1.

 Most respondents were female (*n*=94; 55.9%) (Table S2), with the largest age group being 25–35 years (*n*=51; 22.7%). Those over 60 years were significantly underrepresented (*n*=17; 7.6%) (Table S3).

Analysis revealed significant regional differences in age distribution, χ²(10, *N*=168)=22.674, *p*=0.012, with a 99% Monte Carlo confidence interval of (0.009–0.014): 55.1% of respondents (27 of 49) in Italy were significantly younger than participants from the other regions, being under the age of 25 (Figure S2).

 Most participants had some level of college education, with bachelor’s (*n*=43; 25.7%) and master’s (*n*=53; 31.5%) degrees being the most common (Figure S3; Table S4). Occupational profiles (Figure S4) are provided in the supporting information. 45% of respondents were new to whale-watching (Table S5). Regarding selection factors, 62% of respondents considered ticket price important, 63% were influenced by positive customer reviews, and 49% valued whether the operator was affiliated with a recognized conservation initiative or actively engaged in biodiversity protection efforts. Furthermore, 89% of respondents highlighted the importance of adhering to guidelines when approaching whales, 79% valued opportunities to learn about marine biodiversity conservation, and 63% considered valuable learning about environmental DNA (eDNA).

**Fig. 2:**
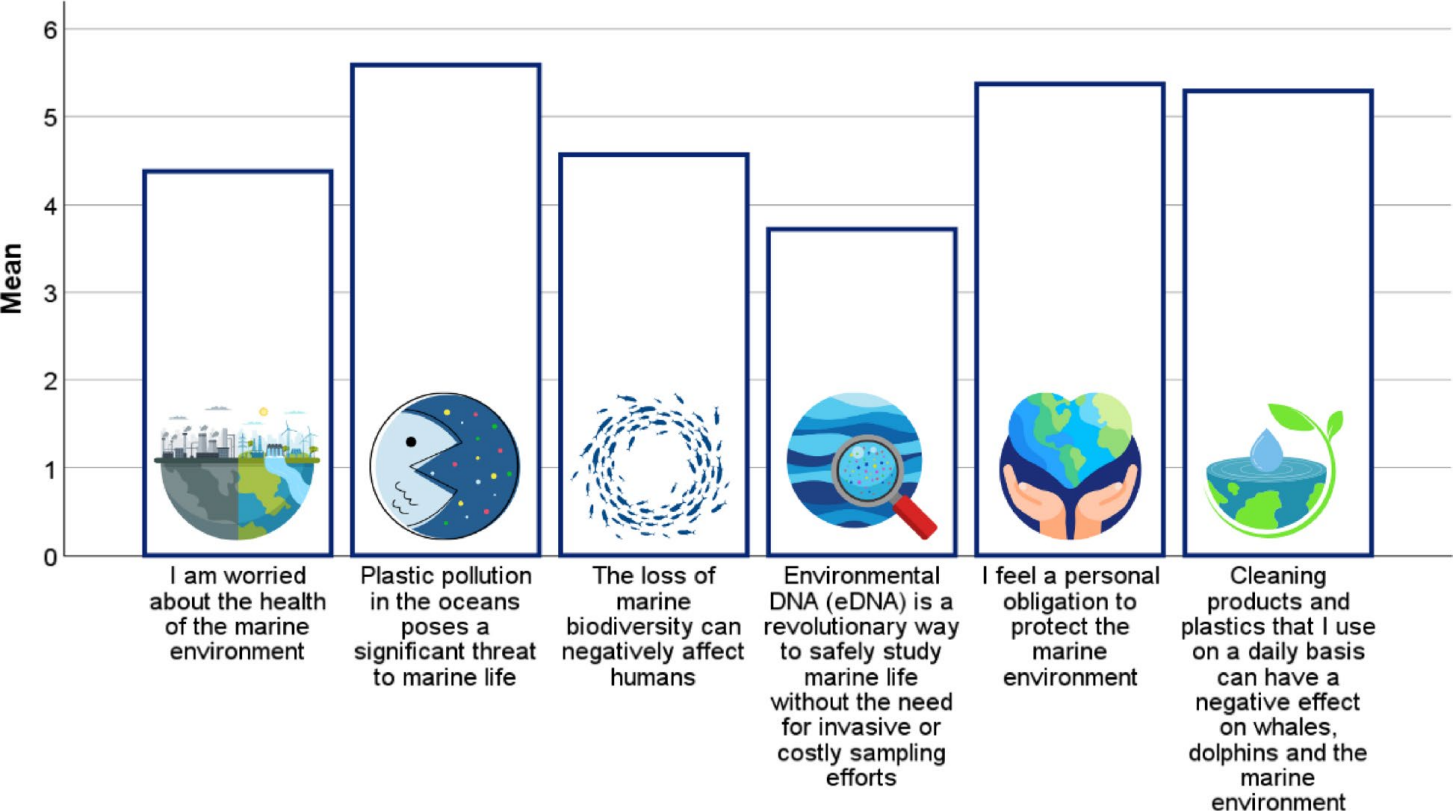
Average level of agreement with statements on marine conservation (*n*=168).

Almost all participants, 96.9% of respondents (216 of 223), expressed concern about the health of the marine environment. Similarly, 97.3% (217/223) agreed that ocean plastic pollution significantly threatens marine life, and that losing marine biodiversity negatively affects humans. Regarding research methods, 87.9% (196/223) considered environmental DNA (eDNA) a revolutionary approach to safely study marine life without invasive or costly sampling. A high proportion (95.5% or 213/223) felt a personal obligation to protect the marine environment, and 91.0% (203/223) agreed that daily cleaning products and plastics may have a negative impact on marine species (Fig. 2).

**Fig. 3:**
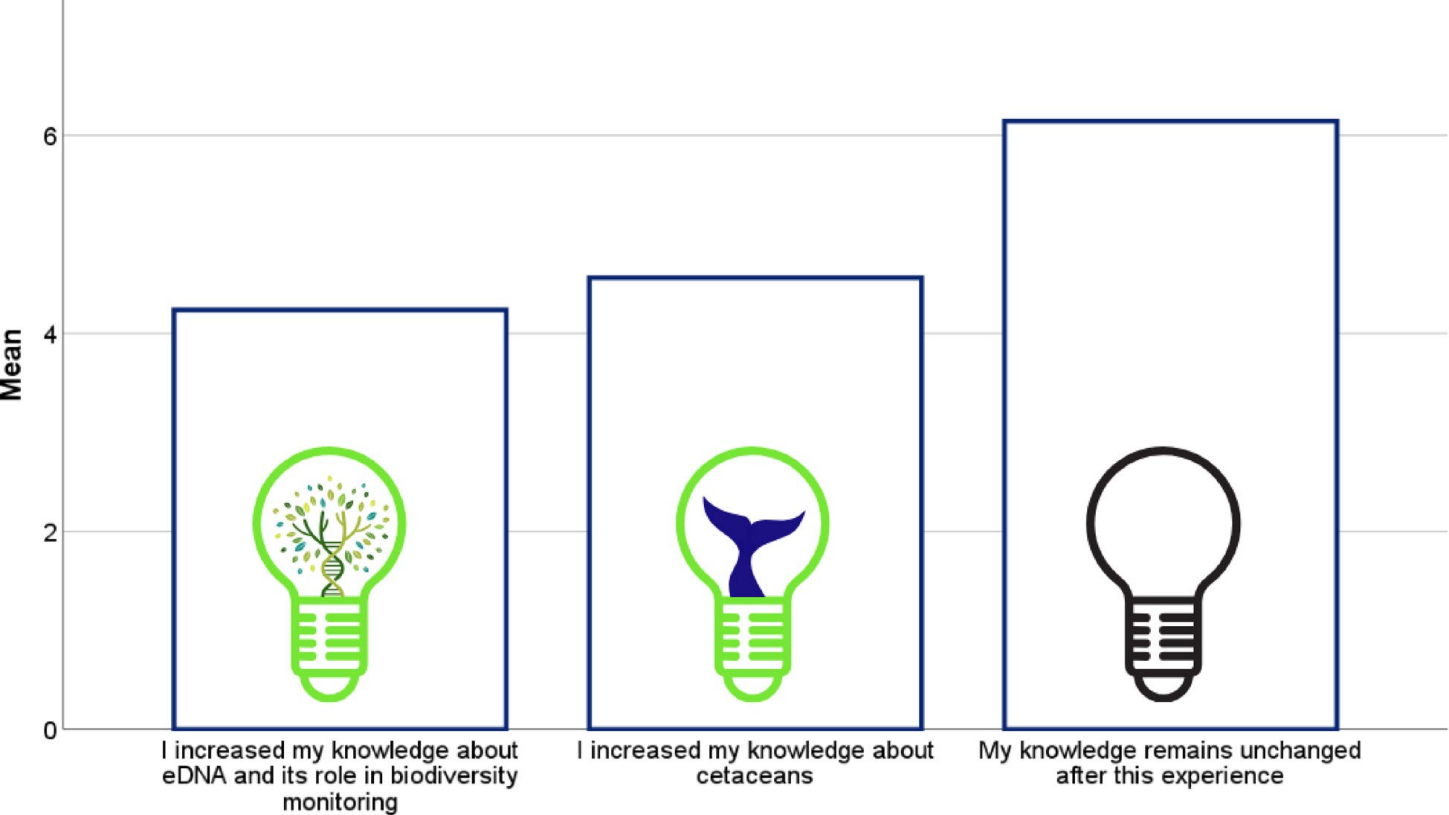
Mean scores for self-reported knowledge gains on eDNA, cetaceans, and perceived absence of learning following the whale-watching experience(*n*=168).

Concerning the statement “I increased my knowledge of eDNA and its role in biodiversity monitoring”, 200 out of 224 participants (89.3%) reported an increase in their knowledge. Statistical analyses revealed no significant differences in eDNA knowledge between the three study areas (p=0.133). Similarly, for the statement “I increased my knowledge about cetaceans,” 208 out of 222 participants (93.7%) reported an increase in their knowledge. In contrast, only 26 out of 223 respondents (11.7%) agreed that “My knowledge remains the same after this experience” (Fig. 3).

**Fig. 4:**
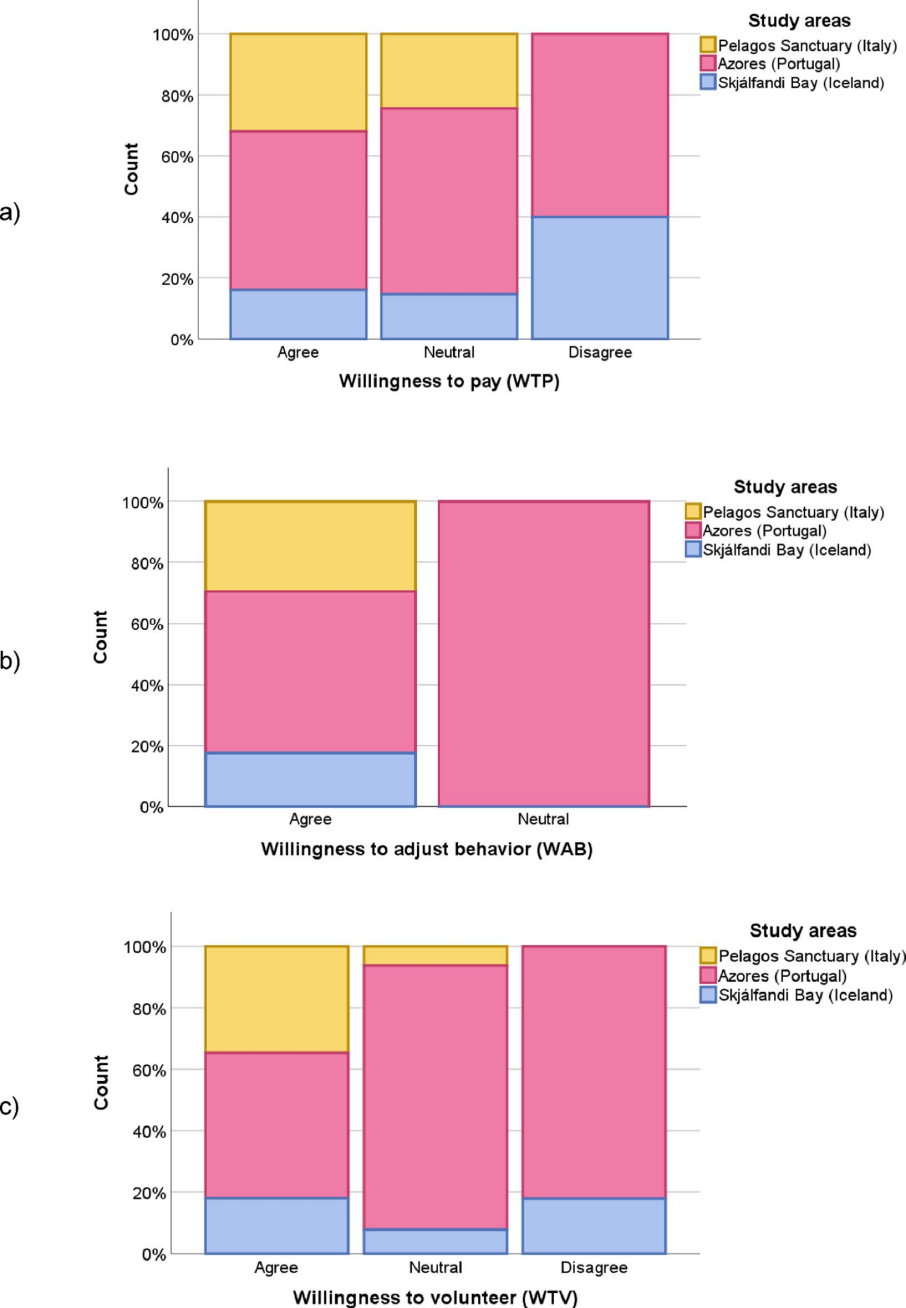
Stacked bar chart of (**a**) willingness to pay (WTP); b) willingness to adjust behavior (WTAB) and c) willingness to volunteer (WTV) for marine conservation (*n*=168).

In terms of commitment, 79.4% (177/223) were willing to fund marine conservation (Fig. 4a), 98.2% (217/221) were ready to change their behavior if it is necessary to protect the marine environment (Fig. 4b), and 84.3% (188/223) were open to volunteering to support marine conservation, such as data collection (Fig. 4c). A positive Spearman correlation (*r*=0.256, *p*<0.001) was observed between participants’ self-reported sense of personal responsibility for marine protection and their stated willingness to donate to conservation initiatives (Q9).

A subsequent Chi-Square Test of Independence revealed that participant willingness to volunteer varied by tour provider (χ²(4)=18.304, *p*=0.001). The highest proportion of volunteers was observed among Italian participants (97.0%), followed by Iceland (91.9%), and then the Azores (74.8%).

Furthermore, FA allowed us to identify the components of the willingness to financially support marine conservation. The rotated Varimax solution revealed distinct factors (varifactors) reflecting environmental awareness, participation in conservation activities, interest in eDNA, and personal conservation behaviors (Table 1). Prior to performing the factor analysis, sampling adequacy was verified. The Kaiser–Meyer–Olkin (KMO) measure was 0.795, indicating a satisfactory level of adequacy, and Bartlett’s test of sphericity was significant (χ²=896.670, df=171, *p*<0.001), confirming that the data were suitable for factor analysis.

**Table 1:** Rotated component matrix from PCA of survey variables (*n*=168).

	Component							
	1	2	3	4	5	6	7	8
Ticket Price								0.921
Affiliation with conservation group			0.673					
Customer ratings on online platforms							0.864	
Knowing that the boat is following guidelines			− 0.732				− 0.402	
Being as close as whales as possible					0.888			
Opportunity to learn about marine biodiversity conservation programs						0.867		
Opportunity to learn about eDNA and its role in biodiversity monitoring		0.552	0.498					
I am worried about the health of the marine environment	0.718							
Plastic pollution in the oceans poses a significant threat to marine life	0.823							
The loss of marine biodiversity can negatively affect humans	0.845							
Environmental DNA (eDNA) is a revolutionary way to safely study marine life without the need for invasive or costly sampling efforts		0.663						
I feel a personal obligation to protect the marine environment		0.560						
Cleaning products and plastics that I use on a daily basis can have a negative effect on marine biodiversity								
I am willing to contribute financially to support marine conservation efforts				0.557				
I am willing to adjust my behavior if it is necessary to protect the marine environment	0.652							
I am willing to actively participate by volunteering to support marine conservation, such as data collection		0.579		0.456				
I increased my knowledge about environmental DNA (eDNA) and its role in biodiversity monitoring		0.730						
I increased my knowledge about cetaceans		0.702						
My knowledge remains unchanged after this experience				0.711				
Extraction Method: Principal Component Analysis. Rotation Method: Varimax with Kaiser Normalization.^a^								

^a^Rotation converged in 38 iterations.

**Fig. 5:**
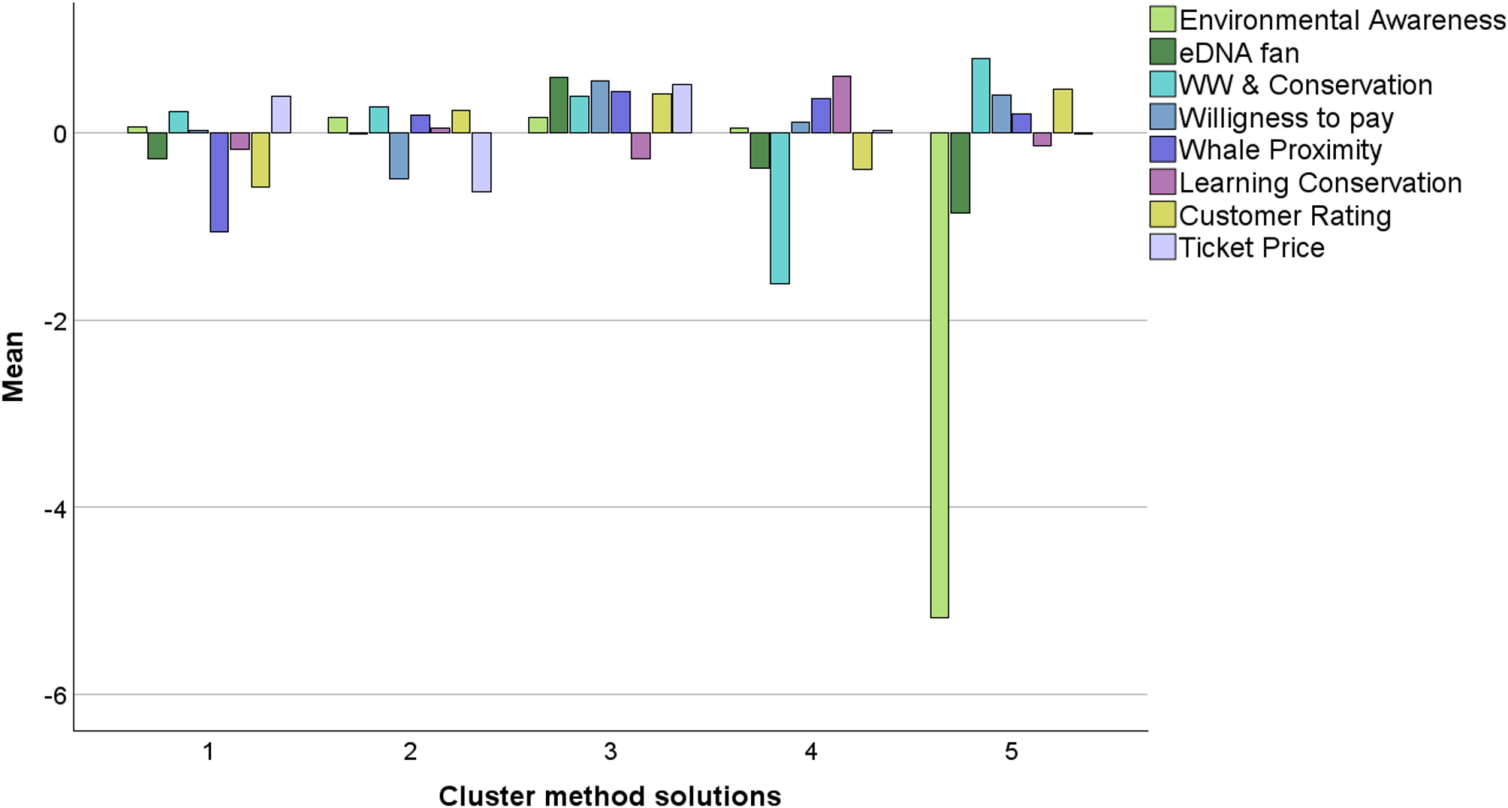
Mean values of survey factors across clusters determined by ward method. x-axis (cluster method solutions): This likely represents different clusters or groupings derived from Ward’s method. Each numbered point (1, 2, 3, etc.) corresponds to a distinct cluster solution, where the data points or participants have been grouped based on similar characteristics or behaviors. Y-axis (Mean): This represents the mean value of the variables being analyzed within each cluster. Each variable (Environmental Awareness, eDNA fan, etc.) has a mean score for the corresponding cluster, which indicates how that variable contributes to or characterizes the cluster (*n*=168).

**Fig. 6:**
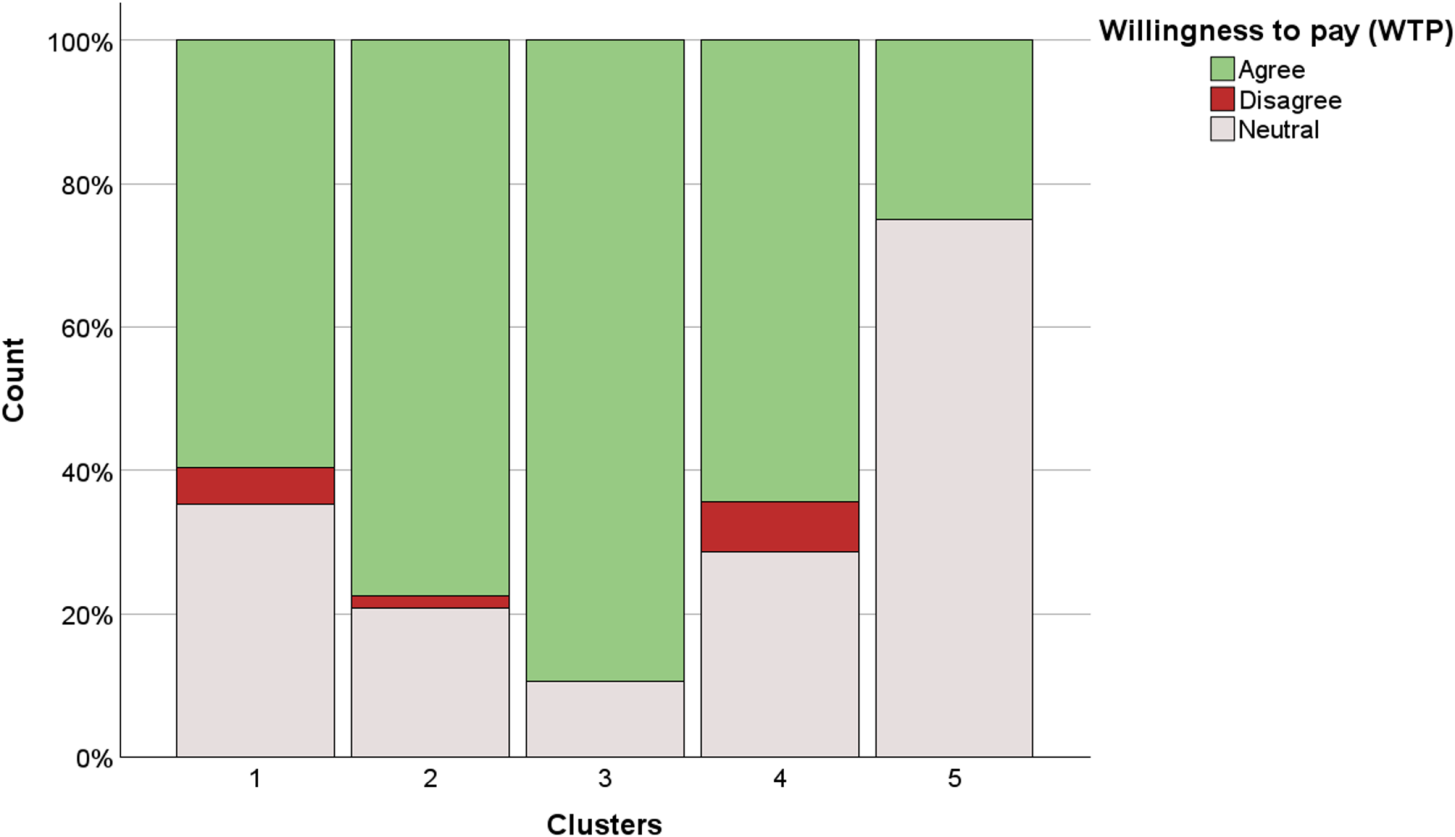
Willingness to pay (WTP) for marine conservation across participant clusters (*n*=168).

Figure 6 presents the distribution of responses to the WTP question for marine conservation, grouped by attitudinal cluster (χ²=10.5; df=3; *p*<0.005). Responses are categorized as follows: “Agree” (green), “Neutral” (blue), and “Disagree” (red). Cluster 3 exhibits the highest proportion of agreement (89.36%), followed closely by cluster 2 (77.51%) and cluster 4 (64.29%). Cluster 4, on the other hand, exhibited the highest percentage of disagreements (7%). Cluster 5 is characterized by predominantly neutral responses, accounting for 75% of the total. No participants expressed disagreement, while a minor proportion expressed agreement, accounting for 25% of the responses.

WTP levels were categorized into low (≤ 30%), moderate (30–65%), and high (> 65%) based on the internal distribution of cluster responses (Table 2).

**Table 2:** Summary of the five respondent clusters identified through HCA, with corresponding WTP levels (*n*=168).

Cluster	Gender	Age	Educational level	WW experience	Interest in eDNA	Conservation learning	WTP
1	Mostly female	19–35	High (University)	Repeaters (2–10+ times)	Low	Low	Moderate (59.60%)
2	Mostly male	Mixed age	High (University)	Beginners	Moderate	Moderate	High (77.51%)
3	Mostly female	Age mixed	High (University)	Repeaters (2–10+ times)	High	Moderate	High (89.36%)
4	Mostly female	36–59	High (University)	Beginners	Low	Moderate	Moderate (64.29%)
5	Mostly male	Under 25	Low (Secondary school)	Beginners	Low	Low	Low (25%)

FA components provided an input data matrix for HCA enabling categorization of respondents into five distinct groups based on shared motivations and behaviours. The five clusters displayed the following sociodemographic and experiential profiles (Table 2; Fig. 5):

Cluster 1 was predominantly composed of females (Figure S5b) and young adults aged 19 to 35 (Figure S5c). The group displayed high educational attainment, with most holding bachelor’s or master’s degrees, and a few participants even holding a Ph.D. (Figure S5d). Many had prior experience with whale-watching, having participated in such activities multiple times (Figure S5e). Despite their academic background and previous exposure to marine ecotourism, the cluster exhibited limited interest in environmental DNA (eDNA) and low environmental awareness, along with only modest engagement with conservation learning. Their WTP for conservation was moderate (59.60%). Motivation among participants of this cluster suggests primarily experiential or recreational orientation. Overall, Cluster 1 reflects a well-educated and experienced group with limited conservation commitment, driven mainly by leisure-related motives.

Cluster 2 was predominantly male (Figure S5b) and included participants of mixed age groups (Figure S5c). Educational levels were high, with many respondents holding master’s or Ph.D. degrees, alongside others with bachelor’s degrees or professional qualifications (Figure S5d). Notably, this cluster was composed entirely of first-time whale-watching participants (Figure S5e). Participants in this group demonstrated price sensitivity, as reflected in their negative perception of ticket costs. They showed moderate levels of environmental awareness and moderate interest in conservation learning but stood out for their high WTP (77.51%) for conservation-related initiatives. Taken together, Cluster 2 represents a highly educated but novice audience, open to conservation support despite limited previous ecotourism experience.

Cluster 3 was predominantly female (Figure S5b) and consisted primarily of adults aged 25 to 45 (Figure S5c). This group stood out as one of the most highly educated, with a clear predominance of participants holding master’s degrees, followed by Ph.D. and bachelor’s qualifications (Figure S5d). Their whale-watching experience was consistent and substantial, with many reporting two to three prior tours, and a notable presence of highly experienced individuals (Figure S5e). The cluster was also internationally diverse (Figure S5a) and demonstrated moderate to strong interest in conservation learning, alongside positive attitudes toward environmental DNA (eDNA). Participants placed high value on the ethical and educational dimensions of the experience, reflecting a well-informed, engaged, and conservation-minded public. Most notably, this group expressed the highest WTP (89.36%), indicating a strong financial and moral commitment to marine conservation efforts.

Cluster 4 was predominantly female (Figure S5b) and composed mainly of participants aged 36 to 59 (Figure S5c). The group exhibited high educational attainment, with most individuals holding master’s or bachelor’s degrees (Figure S5d). Participants had frequent whale-watching experience, often reporting two to three previous tours, and included several highly experienced individuals (Figure S5e). Although the cluster demonstrated low current environmental awareness, it showed a strong will to learn more about marine conservation, suggesting high potential for educational engagement. This openness to learning, combined with their background and experience, positions the group as a strategically valuable audience for targeted awareness and outreach initiatives. Their WTP was moderate (64.29%), indicating a solid baseline of financial support that could increase with greater environmental engagement.

Cluster 5 was composed predominantly of very young males, mostly under the age of 18 (Figures S5b and S5c). This was the least formally educated group, with the majority having completed only secondary school, and very few holding bachelor’s degrees (Figure S5d). The group had minimal prior exposure to whale-watching, with nearly all participants experiencing it for the first time (Figure S5e). This cluster exhibited e5dry low environmental awareness and limited interest in learning about conservation, suggesting a lack of engagement with environmental topics. Consistently, it also showed the lowest WTP (25%), reflecting minimal financial commitment to marine conservation. Despite these limitations, Cluster 5 represents an important early-stage audience, where targeted educational interventions could play a key role in shaping future environmental attitudes and behaviors.

Logistic regression analysis allowed us to identify significant predictors for the WTP for conservation. The stepwise procedure evaluated four models with up to four predictors (Table 3). The best model was found to be the one with four predictors, having environmental awareness as the strongest predictor, with highly aware participants being almost four times more likely to contribute (OR=3.819, *p*<0.001). Furthermore, interest in eDNA was found to double the likelihood of WTP (OR=2.182, *p*<0.001). Conversely, higher ticket prices were observed to significantly reduce WTP (OR=0.632, *p*=0.014) indicating that an increase in ticket price decreases the odds of participants being willing to pay. Finally, higher educational level, specifically a master’s or PhD, was associated with a 2.6-fold increase in the likelihood of WTP (OR=2.601, *p*=0.014). In the final step (Step 4), which incorporated education, environmental awareness, eDNA interest, ticket price, the model correctly classified 78.4% of the “WTP favourable” cases and 69.0% of the “WTP not in favour” cases, with an overall classification accuracy of 73.7%.

**Table 3:** Results from the Stepwise logistic regression analysis of predictors of the WTP for marine conservation efforts (*n*=168).

	Variables in the equation								
		B	S.E.	Wald	df	Sig.	Exp (B)	95% C.I. for EXP(B)	
								Lower	Upper
Step 1^a^	Environmental awareness	1.035	0.297	12.122	1	< 0.001	2.816	1.572	5.044
	Constant	− 0.046	0.169	0.073	1	0.787	0.955		
Step 2^b^	Environmental awareness	1.337	0.359	13.911	1	< 0.001	3.809	1.886	7.691
	eDNA interest	0.677	0.194	12.215	1	< 0.001	1.968	1.346	2.878
	Constant	− 0.095	0.183	0.269	1	0.604	0.910		
Step 3^c^	Environmental awareness	1.401	0.379	13.644	1	< 0.001	4.059	1.930	8.537
	eDNA interest	0.688	0.199	11.934	1	< 0.001	1.991	1.347	2.942
	**Ticket price**	− 0.454	0.185	6.026	1	0.014	0.635	0.442	0.913
	Constant	− 0.077	0.188	0.169	1	0.681	0.926		
Step 4^d^	Educational level	0.956	0.388	6.079	1	0.014	2.601	1.217	5.562
	Environmental awareness	1.340	0.371	13.060	1	< 0.001	3.819	1.847	7.900
	eDNA interest	0.780	0.206	14.304	1	< 0.001	2.182	1.456	3.270
	Ticket price	− 0.459	0.187	6.011	1	0.014	0.632	0.437	0.912
	Constant	− 0.694	0.316	4.831	1	0.028	0.500		

a. Variable(s) entered on step 1: Environmental Awareness.

b. Variable(s) entered on step 2: eDNA fan.

c. Variable(s) entered on step 3: Ticket Price.

d. Variable(s) entered on step 4: Educational level.

Abbreviations: B = unstandardized regression coefficient; S.E.= Standard Error, which measures the precision of the coefficient (B) estimate; Wald = Wald statistic, calculated as (B/S.E)².

## Discussion

The findings of this study offer comprehensive insights into the socio-demographic characteristics, motivations, and environmental perceptions of whale-watching participants involved in citizen science activities. These elements are interpreted within the broader context of sustainable tourism and marine conservation, illustrating how individual attributes, operator selection criteria, and exposure to scientific content, particularly around cetaceans and environmental DNA (eDNA), collectively shape participants’ environmental awareness, informal learning outcomes, and their engagement with conservation, including behavioral adjustments, volunteering intentions, and willingness to pay (WTP) to support marine protection initiatives.

The demographic profile of participants, which is predominantly composed of women, younger adults, and individuals with higher education levels, aligns with broader trends in environmental attitudes and sustainability research^65–67^. These groups have been found to demonstrate stronger ecological values and greater receptivity to conservation messaging. This phenomenon has been documented in various large-scale citizen science and ecotourism initiatives, including Reef Check^68^ and CIGESMED^69^. These observations suggest a broader trend of sustainability-oriented tourists being demographically concentrated among well-educated and environmentally engaged individuals. The regional variation observed in this study, particularly about age and educational attainment, further underscores the importance of adapting science communication strategies to specific audience profiles. The efficacy of such strategies in enhancing accessibility and resonance has been demonstrated, thereby amplifying the impact of conservation messaging and mitigating participation biases^70,71^.

The predominance of first-time whale watchers in our sample indicates that marine ecotourism often provides people’s first direct encounter with cetaceans and their habitats. Such formative encounters play a crucial role in shaping environmental attitudes, particularly when supported by structured interpretation and exposure to conservation narratives^72,73^. On the other side, frequent participants exhibited a heightened awareness of ethical concerns, including minimizing disturbance and prioritizing responsible operators^74^. This finding aligns with the findings of previous research, which suggests that repeated engagement with nature-based tourism strengthens emotional and cognitive connections to marine ecosystems, deepens conservation values, and fosters sustained behavioral change^75–77^).

Recent research provides valuable insights into the cultural, experiential, and technological determinants of financial and behavioral engagement in marine conservation. Ressurreição et al^78^. demonstrated that willingness to pay for marine biodiversity protection varies substantially across European countries, with higher values reported in nations where environmental culture and ecological familiarity are more established. These findings underscore the importance of considering sociocultural baselines when designing conservation finance mechanisms. Dean et al^79^. further emphasized that experiential learning through citizen science not only fosters emotional engagement but also translates into increased support for marine protection and a higher likelihood of adopting new pro-environmental behaviors, especially when participation elicits surprise, reflection, and emotional responses. These findings underscore the pivotal role of emotional connection and familiarity with the marine environment in fostering public support for conservation initiatives. A similar phenomenon has been observed among coastal businesses in Hawaii, where the propensity to invest in coral restoration was not predominantly associated with a distinct perception of economic gain. Instead, it was found to be more closely linked to intrinsic motivations, cultural identity, and the aspiration to uphold a favorable reputation within the community (Carlson et al., 2025). These findings lend support to the notion that conservation engagement can be effectively mobilized through ethical, emotional, and social drivers, thereby complementing the results of our own study on the role of environmental awareness and personal responsibility among whale-watching participants.

The selection of operators by participants was predominantly influenced by economic factors and online reviews. While price frequently functioned as an initial screening tool, particularly in highly competitive destinations such as the Azores or Iceland, online reviews and peer-generated content strongly influenced final decisions. The presence of high ratings and detailed imonials served to enhance the credibility of the operators, particularly when past participants emphasized respectful wildlife interactions or quality interpretation. These outcomes mirror those of tourism research highlighting the interplay between affordability and perceived ethical value in shaping consumer preferences^80–83^.

The environmental credentials of the proposed experience also played a notable role in the selection process. Many participants expressed a preference for whale-watching operators that are visibly engaged in conservation initiatives, such as collaborations with marine researchers, eco-certifications like the Blue Flag, or actions like reducing plastic waste and using biodegradable cleaning products. These efforts were not merely appreciated as ethical gestures but were seen as enhancing the overall value of the experience. Nevertheless, the efficacy of certification programs is contingent upon their visibility and credibility. A body of research has demonstrated that tourists frequently exhibit a lack of awareness or understanding regarding eco-labels. This deficiency impedes the efficacy of these labels, unless they are accompanied by clear and proactive communication from tour providers^84,85^. Initiatives such as Ice Whale in Iceland illustrate how certification can be reinforced through codes of conduct and educational messaging that establish responsible tourism as both meaningful and high-quality^83^. Further evidence from Spain corroborates the findings, demonstrating that a significant proportion of whale-watching tourists exhibit a clear preference for operators that demonstrate social and environmental responsibility, particularly those that are committed to best practices and innovation in the field of sustainability^86^.

Furthermore, the participants exhibited a profound sense of concern regarding the marine environmental threats that were frequently associated with a personal sense of responsibility. Whale-watching has emerged as a powerful vehicle for ecological education, particularly when scientific elements like environmental DNA (eDNA) are integrated into the experience. This tool was widely regarded as non-invasive, innovative, and accessible, enhancing both the legitimacy of conservation narratives and public curiosity about marine science^79,87–89^. Recent advances in protocol development, specifically tailored to whale-watching contexts, have further demonstrated the efficacy of integrating eDNA sampling into tours without compromising detection quality^90^. Concurrently, inter-laboratory ring s have validated the reproducibility of cetacean eDNA assays across diverse extraction methodologies, thereby reinforcing the dependability of results obtained through participatory methodologies in real-world marine tourism operations^91^.

This study underscores how regional differences in whale-watching formats and people engagement approaches can shape not only the structure of the experience but also the composition and expectations of participating audiences. WTP strategies should be meticulously designed based on the local tourism dynamics and modes of engagement. This study methodically compared WTP patterns across three distinct WW models, thereby providing preliminary insights into the alignment of economic support for biodiversity monitoring and conservation with the specific structure and audience engagement approach. Despite the constrained sample size, which precluded the attainment of complete statistical significance for the observed differences among participant segments, this analysis corroborates the hypothesis that participant and attitudinal segmentation vary across regions.

In conclusion, these findings underscore the strategic importance of integrating tourism, science education, and individual empowerment within comprehensive conservation agendas. When supported by reliable citizen science practices, such as eDNA and effective communication, whale-watching evolves beyond its recreational dimension, becoming a significant platform for environmental literacy, stewardship, and public engagement. In this context, marine ecotourism emerges not only as a site of enjoyment, but also as a scalable, participatory model for advancing the objectives of global frameworks such as the EU Mission “Restore our Ocean and Waters” and the Global Biodiversity Framework (European Commission, 2020; UNEP, 2021). Initiatives such as the combination of eDNA and citizen engagement are increasingly recognized as strategic tools for fostering inclusive governance in emerging ocean policies^92^. Future research should investigate the persistence of behavioral change over time and assess the scalability of this model in diverse governance and tourism contexts.

## Conclusion

This study assessed the effectiveness of integrating environmental DNA (eDNA) sampling and citizen science into whale-watching as a tool for promoting marine conservation. Conducted across three European regions, the survey revealed significant increases in environmental awareness, perceived personal responsibility, and willingness to engage in conservation through behavioural changes, financial support and volunteering. Environmental awareness, interest in eDNA, and higher education levels emerged as significant predictors of willingness to pay for conservation-oriented experiences. Conversely, higher ticket prices were associated with a reduction in financial commitment. These findings show that short, non-invasive educational activities can promote pro-environmental engagement. Tailored communication for different socio-demographic groups can enhance these effects and improve inclusivity. By linking citizen science, informal environmental education, and financial engagement, this study provides actionable insights for tourism operators, conservation practitioners, and environmental managers. The proposed approach may contribute to biodiversity monitoring, support public engagement, and promote the long-term sustainability of conservation efforts. The eWHALE experience incorporates participatory models within ecotourism, aligning with policy frameworks such as the EU Biodiversity Strategy for 2030, the United Nations Sustainable Development Goals, and the Global Biodiversity Framework^93,94^.

## Supplementary Information

Below is the link to the electronic supplementary material.

Supplementary Material 1

Supplementary Material 2

## Data Availability

The anonymized questionnaire dataset and associated analysis scripts that support the findings of this study are available from the corresponding author upon reasonable request. The dataset contains only aggregated, non-identifiable responses (age class, education level, etc.) and can be shared subject to data use agreement.
