# Supplementary material for: Willingness to engage in marine conservation through eDNA-informed citizen science on whale-watching platforms
Source: Sci Rep. 2025 Nov 25;15:41983. doi: 10.1038/s41598-025-26209-4 (PMC12647838; doi:10.1038/s41598-025-26209-4)
Supplement: Supplementary file 1 — Supplementary Material 1 [file 41598_2025_26209_MOESM1_ESM.pdf]

eWHALE SURVEY

By participating in this voluntary questionnaire, you consent to the sharing of your answers with members of the eWHALE team. Your responses are collected for scientific purposes and will be treated with confidentiality and anonymity. Only aggregated data will be shared. Thank you for your valuable input.

* 1. Which country are you currently practicing whale watching in?

- ◯ Iceland (North Sailing)
- ◯ Italy (Tethys)
- ◯ Portugal (CW Azores)

* 2. Country of residence

* 3. How old are you?

- ◯ Under 18
- ◯ 19-24
- ◯ 25-35
- ◯ 36-45
- ◯ 46-59
- ◯ Over 60

* 4. What gender do you identify with?

- ◯ Female
- ◯ Male
- ◯ Non-Binary

* 5. What is your highest grade of education?

- ◯ No formal education
- ◯ Primary school
- ◯ Secondary school
- ◯ Bachelor's degree
- ◯ Master's degree
- ◯ Doctorate (Ph.D.)
- ◯ Professional certifications or specialized training

* 6. Which of the following best describe your job? (More than 1 option is possible)

- ☐ Academic/Research
- ☐ Information Technology
- ☐ Healthcare/Medical
- ☐ Business/Finance
- ☐ Marketing/Advertising
- ☐ Education/Teaching
- ☐ Arts/Entertainment
- ☐ Engineering/Technology
- ☐ Nonprofit/NGO
- ☐ Marine/Nature Related
- ☐ Government/Public Service
- ☐ Communication/Media
- ☐ Legal/Law
- ☐ Not a worker
- ☐ Other

* 7. How many whale watching experiences have you had? (Include this one in your response!)

- ◯ 1st time
- ◯ 2nd time
- ◯ 3rd time
- ◯ 3 - 10 times
- ◯ > 10 times

* 8. **Could you please rate how important the following aspects are to you when choosing a whale watching company?**

	Very important	Moderately Important	Slightly Important	Neutral	Slightly Not Important	Moderately Not Important	Not [illegible] Impo[illegible]
Ticket Price	○	○	○	○	○	○	○
Affiliation with conservation group (e.g. eWHALE)	○	○	○	○	○	○	○
Customer ratings on online platforms (TripAdvisor, google...)	○	○	○	○	○	○	○
Knowing that the boat is following guidelines (e.g. safe distance from whales)	○	○	○	○	○	○	○
Being as close to the whales as possible	○	○	○	○	○	○	○
Opportunity to learn about marine biodiversity conservation programs (e.g. eWHALE) and how to get involved	○	○	○	○	○	○	○
Opportunity to learn about eDNA and its role in	○	○	○	○	○	○	○

	Very important	Moderately Important	Slightly Important	Neutral	Slightly Not Important	Moderately Not Important	Not [illegible] Impo[illegible]
biodiversity monitoring							

9. **Could you please indicate to what extent you agree with the following statements regarding the marine environment?**

	Strongly Agree	Moderately Agree	Slightly Agree	Neutral	Slightly Disagree	Moderately Disagree	Strongly Disagree
I am worried about the health of the marine environment	○	○	○	○	○	○	○
Plastic pollution in the oceans poses a significant threat to marine life	○	○	○	○	○	○	○
The loss of marine biodiversity can negatively affect humans	○	○	○	○	○	○	○
Environmental DNA (eDNA) is a revolutionary way to safely study marine life without the need for invasive or costly sampling efforts	○	○	○	○	○	○	○
I feel a personal obligation to protect the marine environment	○	○	○	○	○	○	○
Cleaning products and	○	○	○	○	○	○	○

	Strongly Agree	Moderately Agree	Slightly Agree	Neutral	Slightly Disagree	Moderately Disagree	Strongly Disagree
plastics that I use on a daily basis can have a negative effect on whales, dolphins and the marine environment							
I am willing to contribute financially to support marine conservation efforts	○	○	○	○	○	○	○
I am willing to adjusting my behaviour if it is necessary to protect the marine environment	○	○	○	○	○	○	○
I am willing to actively participate by volunteering to support marine conservation, such as data collection	○	○	○	○	○	○	○

10. **Could you please indicate to what extent you agree with the following statements regarding this whale watching/citizen science experience?**

	Strongly Agree	Moderately Agree	Slightly Agree	Neutral	Slightly Disagree	Moderately Disagree	Strongly Disagree
I increased my knowledge about environmental DNA (eDNA) and its role in biodiversity monitoring	○	○	○	○	○	○	○

	Strongly Agree	Moderately Agree	Slightly Agree	Neutral	Slightly Disagree	Moderately Disagree	Strongly Disagree
I increased my knowledge about cetaceans	◯	◯	◯	◯	◯	◯	◯
My knowledge remains unchanged after this experience	◯	◯	◯	◯	◯	◯	◯

DONE

Powered by
SurveyMonkey®
See how easy it is to create surveys and forms.

Privacy & Cookie Notice
