# Supplementary material for: Willingness to engage in marine conservation through eDNA-informed citizen science on whale-watching platforms
Source: Sci Rep. 2025 Nov 25;15:41983. doi: 10.1038/s41598-025-26209-4 (PMC12647838; doi:10.1038/s41598-025-26209-4)
Supplement: Supplementary file 2 — Supplementary Material 2 [file 41598_2025_26209_MOESM2_ESM.docx]

**SUPPLEMENTARY MATERIAL S2**

**Willingness to engage in marine conservation through eDNA-informed citizen science on whale-watching platforms**

Figure S1 provides a visual overview of the study design, research question, study areas and key findings. The left panel presents the research question and outlines the integration of whale-watching citizen science with eDNA to foster public engagement in marine conservation. The center panel maps the three European case studies and summarises participation and self-reported learning gains from on-board activities. The right panel shows the main outcome: 80% of respondents were willing to pay for marine conservation, with higher environmental awareness and education increasing willingness, and higher ticket prices reducing it.


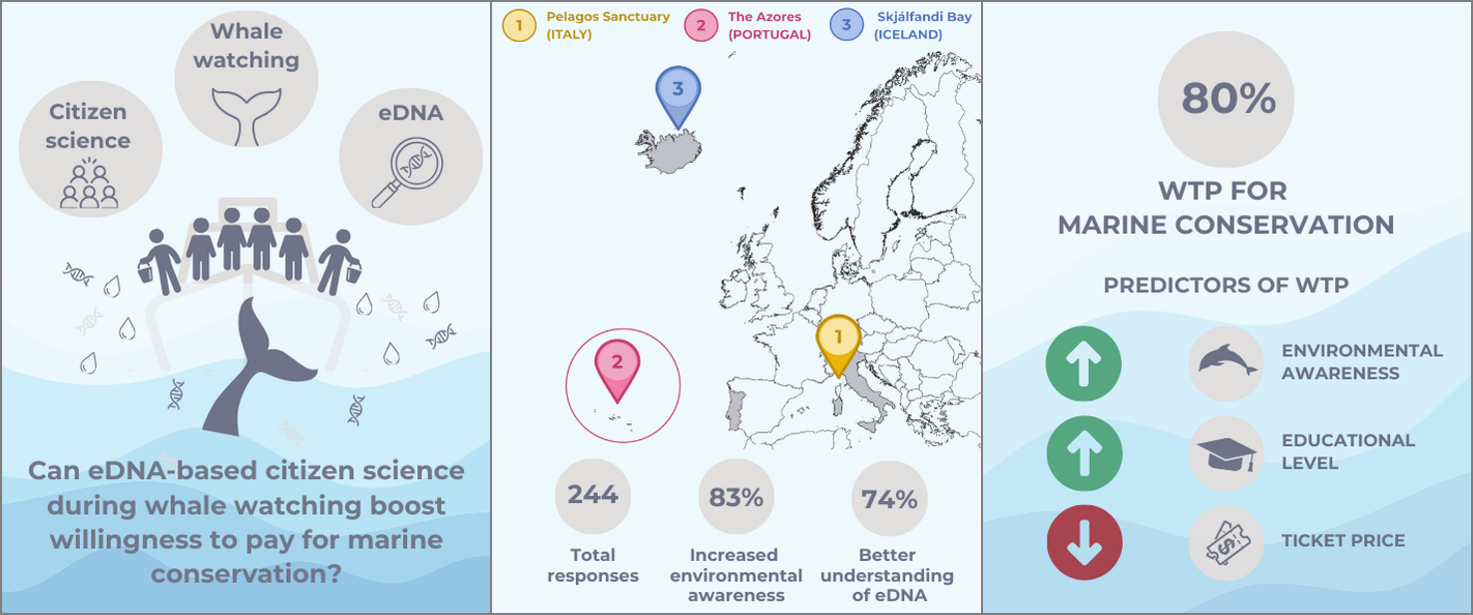


***Figure S1.*** *Graphical summary of the study. Left: conceptual link between whale-watching, citizen science and eDNA, framing the research question on whether eDNA-based citizen science can increase willingness to pay (WTP) for marine conservation. Centre: study locations in the Pelagos Sanctuary (Italy), the Azores (Portugal) and Skjálfandi Bay (Iceland), with sample size and immediate learning outcomes (244 responses; 83% reported increased environmental awareness; 74% reported improved understanding of eDNA). Right: overall WTP for marine conservation was 80%; key predictors were environmental awareness and educational level (positive) and ticket price (negative).*

Table S1 reports the cross-tabulation of participants’ highest completed education by age class for each study site and for the overall sample.

Age classes were defined a priori as Under 18, 19–24, 25–35, 36–45, 46–59 and Over 60. Education levels were harmonized across national systems and mapped to six categories: Primary School, Secondary School, Professional certifications, BSc, MSc and PhD.

Cell entries are unweighted counts (row frequencies); site-level and overall totals are shown. The cross-tabulation includes only respondents with non-missing values for both age and education. Therefore, the sample size for this table is n=168, which is lower than the full survey N due to missing or “prefer not to say” responses in either field.

***Table S1.*** *Demographic information about whale-watching tourists, showing row frequencies (n=168).*

|  | | | **Primary School** | **Secondary School** | | **Professional certifications** | **BSc** | **MSc** | **PhD** | **Total** |
| --- | --- | --- | --- | --- | --- | --- | --- | --- | --- | --- |
|  |  |  | **n** | **n** | **n** | | **n** | **n** | **n** | **n** |
| **Pelagos Sanctuary (Italy)** |  | **Under 18** | 2 | 13 | 0 | | 0 | 0 | 0 | 14 |
|  |  | **19-24** | 0 | 9 | 0 | | 3 | 1 | 0 | 13 |
|  |  | **25-35** | 0 | 0 | 0 | | 2 | 3 | 0 | 5 |
|  |  | **36-45** | 0 | 0 | 1 | | 2 | 2 | 1 | 6 |
|  |  | **46-59** | 0 | 1 | 0 | | 1 | 4 | 1 | 7 |
|  |  | **Over 60** | 0 | 0 | 3 | | 1 | 0 | 0 | 4 |
|  |  | **Total** | 2 | 23 | 4 | | 8 | 11 | 2 | 49 |
| **Azores (Portugal)** |  | **Under 18** | 4 | 4 | 0 | | 0 | 0 | 0 | 8 |
|  |  | **19-24** | 0 | 7 | 0 | | 5 | 0 | 0 | 11 |
|  |  | **25-35** | 0 | 1 | 1 | | 9 | 10 | 2 | 22 |
|  |  | **36-45** | 0 | 2 | 1 | | 4 | 11 | 3 | 19 |
|  |  | **46-59** | 0 | 3 | 3 | | 4 | 9 | 4 | 23 |
|  |  | **Over 60** | 0 | 1 | 1 | | 5 | 1 | 0 | 8 |
|  |  | **Total** | 4 | 17 | 5 | | 26 | 31 | 9 | 92 |
| **Skjálfandi Bay (Iceland)** |  | **Under 18** | 0 | 2 | 0 | | 0 | 0 | 0 | 2 |
|  |  | **19-24** | 0 | 1 | 0 | | 5 | 1 | 0 | 6 |
|  |  | **25-35** | 0 | 0 | 2 | | 2 | 4 | 2 | 9 |
|  |  | **36-45** | 0 | 1 | 0 | | 1 | 3 | 1 | 5 |
|  |  | **46-59** | 0 | 0 | 0 | | 1 | 3 | 0 | 5 |
|  |  | **Over 60** | 0 | 0 | 0 | | 0 | 1 | 0 | 1 |
|  |  | **Total** | 0 | 3 | 2 | | 9 | 11 | 3 | 27 |
| **Total** |  | **Under 18** | 5 | 19 | 0 | | 0 | 0 | 0 | 24 |
|  |  | **19-24** | 0 | 16 | 0 | | 13 | 2 | 0 | 30 |
|  |  | **25-35** | 0 | 1 | 3 | | 13 | 17 | 4 | 37 |
|  |  | **36-45** | 0 | 2 | 1 | | 6 | 16 | 5 | 30 |
|  |  | **46-59** | 0 | 4 | 3 | | 6 | 17 | 5 | 34 |
|  |  | **Over 60** | 0 | 1 | 4 | | 6 | 2 | 0 | 13 |
|  |  | Total | 5 | 42 | 11 | | 43 | 53 | 14 | 168 |

The analysis of gender distribution among participants indicates a notable difference in representation between males and females (Table S2). Due to low frequency (n=4), Non-binary (NB) gender was not included in the analysis. The survey results show that 55.9% of the respondents were female (n=94), while 44.1% were male (n=74). This imbalance was found to be statistically significant (p=0.032). However, there were no significant differences in gender distribution between the three sites (chi-square=0.080; df=2; p=0.961).

***Table S2.*** *Gender distribution of respondents by region, showing the count and percentage of female and male participants across the three study areas and in total (n=167).*

|  | |  | | | | | | | |
| --- | --- | --- | --- | --- | --- | --- | --- | --- | --- |
|  |  | **Pelagos Sanctuary**  **(Italy)** | | **Azores**  **(Portugal)** | | **Skjálfandi Bay (Iceland)** | | **Total** | |
|  |  | **n** | **%** | **n** | **%** | **n** | **%** | **n** | **%** |
|  | **Female** | 28 | 57.6% | 51 | 55.7% | 14 | 53.7% | 94 | 55.9% |
|  | **Male** | 21 | 42.4% | 41 | 44.3% | 12 | 46.3% | 74 | 44.1% |
|  | **Total** | 49 | 100% | 92 | 100% | 27 | 100% | 167 | 100% |

A chi-square test of independence was performed to assess whether age group distributions differed across the three study areas (Figure S2; Table S3). The analysis revealed a statistically significant association between age group and study area (χ²(10)=22.674, p=0.012), indicating that participant age profiles varied across countries. Specifically, Portugal was characterized by a higher representation of participants aged 25–59, Italy showed a greater proportion of younger respondents (under 18 and 19–24), while Iceland displayed a more balanced but numerically smaller distribution across age groups. The linear-by-linear association was also significant (χ²(1)=4.340, p=0.037), suggesting a potential ordinal trend in age distribution across regions.

***
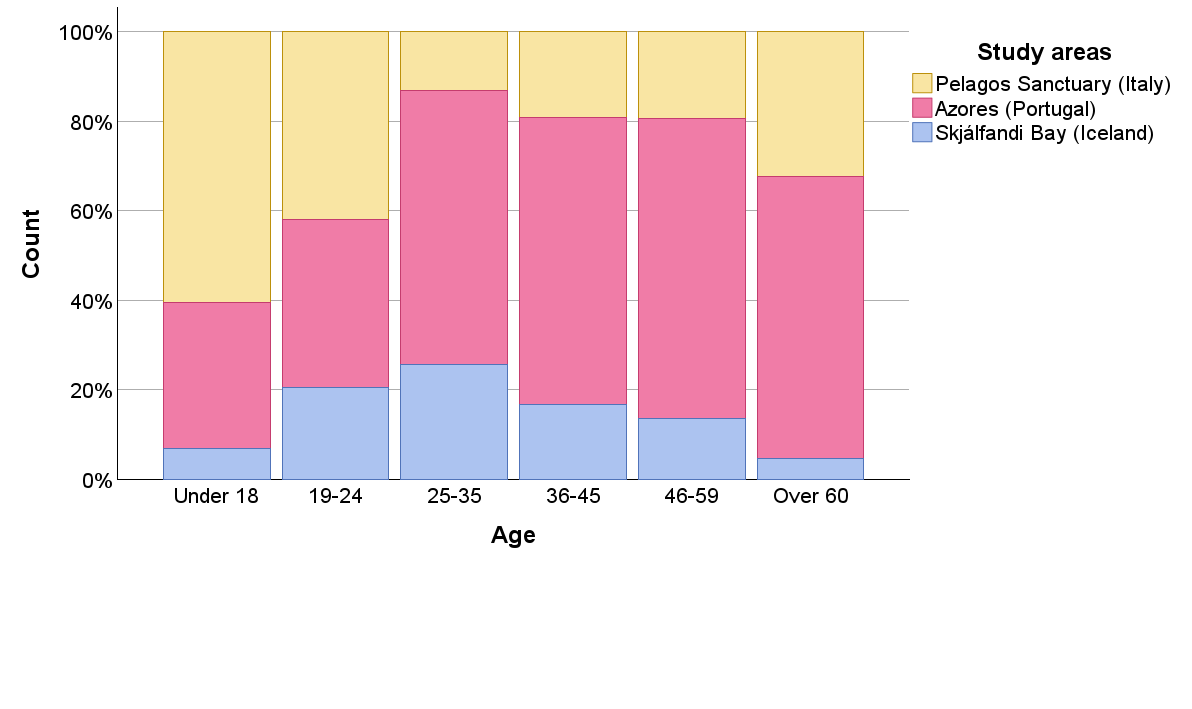
***

***Figure S2.*** ***Age distribution of respondents by region:*** *the stacked bar chart shows the percentage of participants from each entry categorized by age group, ranging from Under 18 to Over 60.*

***Table S3.*** *Age distribution of respondents by study area, showing the number (n) and percentage (%) of participants in each age group (under 18, 19–24, 25–35, 36–45, 46–59, over 60) across the three study areas and in total.*

|  | | **Pelagos Sanctuary**  **(Italy)** | | **Azores**  **(Portugal)** | | **Skjálfandi Bay**  **(Iceland)** | | **Total** | |
| --- | --- | --- | --- | --- | --- | --- | --- | --- | --- |
|  |  | **n** | **%** | **n** | **%** | **n** | **%** | **n** | **%** |
|  | **Under 18** | 14 | 29.6% | 8 | 8.5% | 2 | 5.8% | 24 | 14.2% |
|  | **19-24** | 13 | 26.3% | 11 | 12.4% | 6 | 22.6% | 30 | 18.1% |
|  | **25-35** | 5 | 9.9% | 22 | 24.3% | 9 | 34.3% | 37 | 21.8% |
|  | **36-45** | 6 | 11.9% | 19 | 21.1% | 5 | 18.2% | 30 | 18% |
|  | **46-59** | 7 | 13.6% | 23 | 24.8% | 5 | 16.8% | 34 | 20.2% |
|  | **Over 60** | 4 | 8.6% | 8 | 8.9% | 1 | 2.2% | 13 | 7.7% |
|  | **Total** | 49 | 100% | 92 | 100% | 27 | 100% | 168 | 100% |

The analysis of educational levels of participants showed significant variation between the categories (χ²=107.446, df=5, p<0.001), indicating a non-uniform distribution, with certain groups being more represented than others. Specifically, the most common educational levels were Master's degree (31.5%) and Bachelor's degree (25.7%). Participants with secondary education comprised 25.2% of the sample. Conversely, those with a doctorate (8.1%), primary education (3.1%), and professional certifications or specialized training (6.3%) were underrepresented in the sample (Table S4).

***Table S4.*** *Distribution of participants by highest education level, showing frequency, percent, valid percent, and cumulative percent from primary school to doctoral level.*

|  | | | | | |
| --- | --- | --- | --- | --- | --- |
|  | | **Frequency** | **Percent** | **Valid Percent** | **Cumulative Percent** |
|  | **Primary School** | 5 | 3.1 | 3.1 | 3.1 |
|  | **Secondary School** | 42 | 25.2 | 25.2 | 28.3 |
|  | **Professional certifications** | 11 | 6.3 | 6.3 | 34.6 |
|  | **Bachelor's degree(BsC)** | 43 | 25.7 | 25.7 | 60.4 |
|  | **Master's degree (MsC)** | 53 | 31.5 | 31.5 | 91.9 |
|  | **PhD** | 14 | 8.1 | 8.1 | 100.0 |
|  | **Total** | 168 | 100.0 | 100.0 |  |

The distribution of educational levels among whale-watching participants varied considerably across the three surveyed regions (χ²=29.203, df=10, p<0.001; Figure S3). In particular, secondary education is significantly over-represented in Italy. Further analysis clarifies that this pattern is linked to the younger age profile of Italian participants, many of whom were under 18, rather than indicating lower educational attainment among adults (χ²=71.285, df=25, p<0.001). In all regions, there were relatively few participants with a Ph.D. Those from Iceland (North Sailing) were dominated by individuals with a Master's degree. The Azores (CW Azores) showed a more even distribution between respondents having a Bachelor's and Master's degrees.


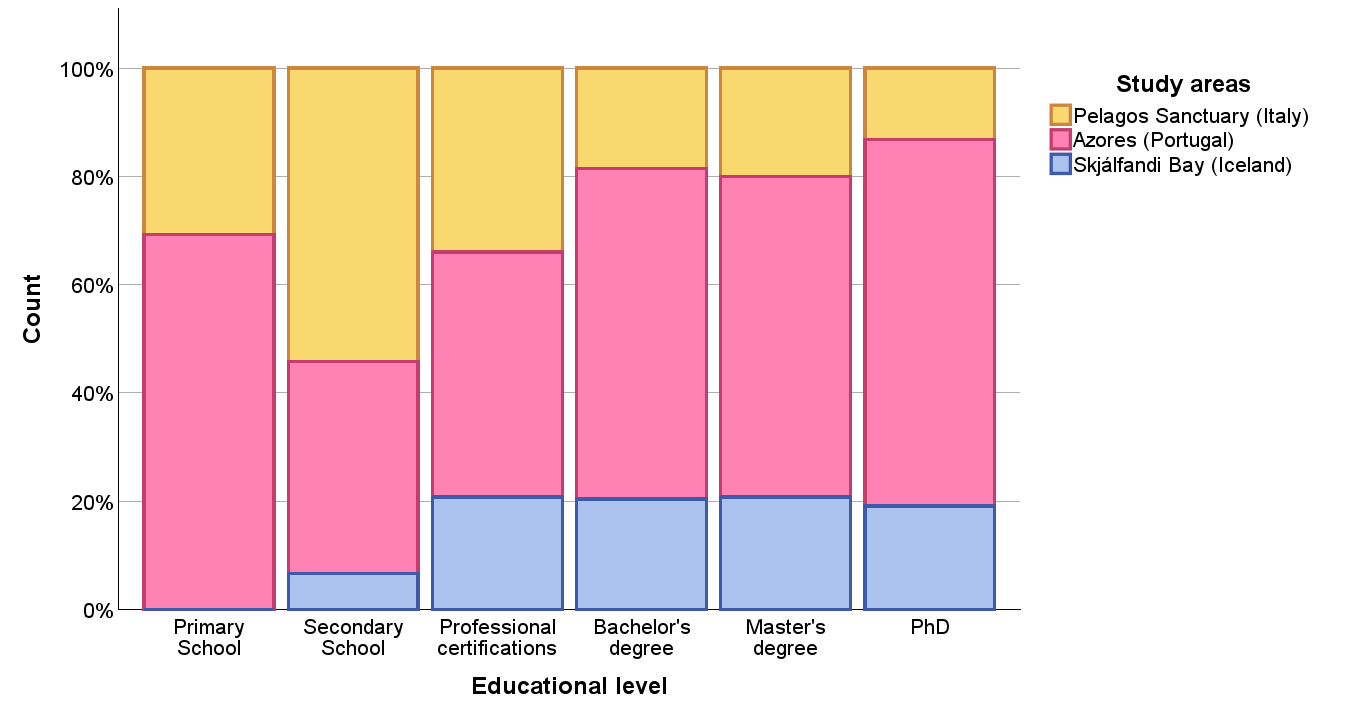


***Figure S3.*** ***Educational level distribution of respondents by region:*** *the stacked bar chart shows the percentage of participants from each entry categorized by their highest level of education, ranging from primary school to PhD level.*

Figure S4 shows the distribution of occupational categories among respondents. The most frequently selected category was "not employed" (19.64%), followed by Healthcare/Medical (18.30%). Academic/Research was reported by 13.39% of respondents, while Business/Financial accounted for 11.61%. Marine/Nature-related fields and Education/Teaching were each selected by 8.04% of respondents. In contrast, sectors such as Nonprofit/NGO (4.46%), Government/Public Service (4.46%), Communication/Media (3.57%), Marketing/Advertising (3.13%), Arts/Entertainment (5.80%), Engineering/Technology (9.82%), Information Technology (6.70%), and Law/Legal (1.34%) were less frequently selected. In addition, 8.48% of respondents selected the "Other" category, reflecting a variety of occupations not included in the predefined options.


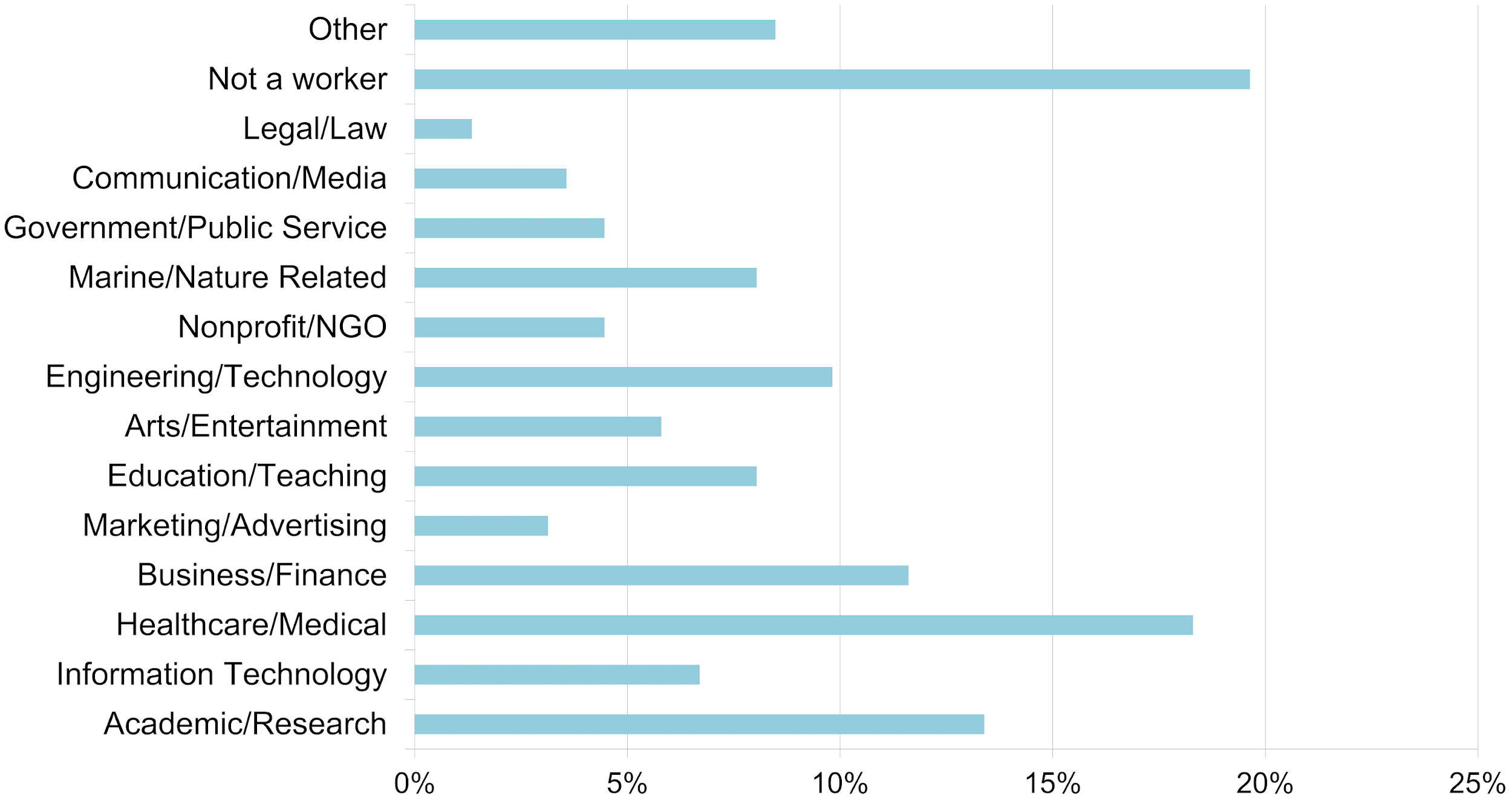


***Figure S4.*** *Distribution of occupational categories among participants.*

Following the demographic questions, participants were asked about the number of whale-watching experiences they had. Analysis revealed that 45.0% of respondents were on their first whale-watching tour, while 25.1% were on their second. In addition, 10.5% had experienced three tours and 12.7% had participated in whale-watching between three and ten tours. A smaller but notable number, 6.7%, had been on more than ten tours (Table S5).

***Table S5.*** *Frequency, percent, valid percent, and cumulative percent of participants based on their number of whale-watching experiences, categorized from "1st time" to "> 10 times".*

| **WW experience** | | | | | |
| --- | --- | --- | --- | --- | --- |
|  | | **Frequency** | **Percent** | **Valid Percent** | **Cumulative Percent** |
|  | **1^st^ time** | 76 | 45.0 | 45.0 | 45.0 |
|  | **2^nd^ time** | 42 | 25.1 | 25.1 | 70.1 |
|  | **3^rd^ time** | 18 | 10.5 | 10.5 | 80.6 |
|  | **3 - 10 times** | 21 | 12.7 | 12.7 | 93.3 |
|  | **> 10 times** | 11 | 6.7 | 6.7 | 100 |
|  | **Total** | 168 | 100 | 100 |  |

In consideration of the responses concerning the significance of various factors that tourists considered when choosing a whale-watching operator, a substantial proportion of respondents indicated that the cost of whale-watching tours was a considerable factor in their selection process of a whale-watching tour provider. The results of the survey revealed that approximately 62% of respondents indicated that ticket price was either "moderately important" or "very important," thereby underscoring its substantial impact on their decision-making processes. Conversely, approximately 19% of respondents indicated that ticket price was less of an issue. The remaining respondents, constituting approximately 19% of the total, expressed a neutral stance on the matter, indicating an absence of a pronounced opinion either in favor of or in opposition to the proposal. When asked about the importance of a whale-watching operator's affiliation with a conservation group, opinions varied considerably.

When asked about the importance of a whale-watching operator's affiliation with a conservation group, opinions varied considerably. A significant proportion of respondents, 49.11%, felt that this affiliation was "very important” in their choice of operator, while 23.21% felt it was “moderately important”. In contrast, around 16% of respondents did not consider membership of a conservation group to be important. Some 11.61% of respondents were neutral on the issue, meaning that it wasn't a deciding factor for them.

The majority of respondents found positive customer reviews on online platforms to be an important factor when considering a whale-watching operator. An overwhelming 62.94% of respondents rated customer reviews as either "moderately important" or "very important". Meanwhile, 14.29% of respondents were neutral on the issue. The remaining 22.77% considered customer reviews to be "not important", showing that for a smaller group, reviews have little influence on their choice of tour operator.

When asked about the importance of following guidelines when approaching a whale, the vast majority of respondents felt it was important (88.84%), while only 6.70% felt it was "not important" and 4.46% were neutral. There were varying responses to the importance of being as close as possible to the whale during a trip, suggesting that while proximity is important to some, many participants prioritize respect for the animal and its natural environment. With 22.77% of respondents stating that proximity was 'not important' and 16.07% remaining neutral, it is likely that a significant proportion of participants value maintaining a respectful distance to avoid disturbing the whale.

A majority of respondents (79.46%) considered the opportunity to learn about marine biodiversity conservation during whale-watching trips important. In contrast, 8.03% of respondents considered this opportunity to be "not important". Meanwhile, 11.16% of respondents were neutral, indicating no strong preference one way or the other. Over half (63.39%) of respondents felt that learning about environmental DNA (eDNA) during their whale-watching tour was important. Only 10.71% of respondents found this aspect "not important", while 18.30% remained neutral, reflecting a general interest in conservation education among participants.

The five respondent clusters showed distinct patterns across key sociodemographic variables. Clear geographical differences emerged across the five clusters in terms of country of origin (Figure S5a). Cluster 1 was the most internationally diverse, with substantial representation from multiple countries including Portugal, Germany, Iceland, Australia, and Austria, as well as notable presence from France, the USA, and other European nations. Cluster 2 also showed a broad international profile, prominently featuring respondents from Austria, Australia, Portugal, and several Northern and Western European countries. Cluster 3 was mainly composed of individuals from Portugal, but also included participants from Germany, the United Kingdom, Iceland, and other countries, reflecting a moderate degree of heterogeneity. Cluster 4 included a wide distribution of nationalities, though Portugal and Iceland were particularly well represented, along with Germany and France. Cluster 5, by contrast, showed the highest degree of national concentration, with an overwhelming majority of participants coming from Portugal, accompanied by smaller proportions from Iceland and Russia. These patterns suggest that while some clusters gathered respondents from a wide range of national backgrounds, others were more nationally homogeneous, which may reflect differences in sampling locations or in the appeal of whale-watching experiences across regions.

Gender differences across clusters were also evident (Figure S5b). While Clusters 1, 3, and 4 were predominantly female, Cluster 2 had a more balanced distribution between males and females. Cluster 5 stood out as the only group with a male majority, highlighting a generational and gendered composition that contrasts with the rest.

Age distributions (Figure S5c) further differentiated the clusters. Cluster 5 consisted almost entirely of very young participants under the age of 18. Cluster 4, on the other hand, was composed primarily of adults between 36 and 59 years old. Clusters 1, 2, and 3 had more heterogeneous age profiles, with a predominance of individuals aged between 19 and 45 years.

Education levels (Figure S5d) revealed that Cluster 4 had the highest academic attainment, with a large proportion of participants holding a Bachelor's or Master’s degree. Cluster 5 showed the lowest educational level, with the majority having only completed secondary or even primary school. Clusters 1, 2, and 3 were more evenly distributed but still skewed toward higher education levels.

Finally, whale-watching experience (Figure S5e) varied significantly across groups. Cluster 2 was composed entirely of first-time whale watchers. Clusters 1 and 3 also included a majority of first-time participants but featured a noticeable share of more experienced individuals, including those who had joined whale-watching tours more than ten times. Cluster 4 emerged as the most experienced group, with nearly 60% of its members having taken part in multiple tours. Cluster 5 was largely composed of participants on their second whale-watching experience but also included a substantial portion of repeat participants, highlighting a mix of emerging and consolidated interest in marine ecotourism.

| **a)** | **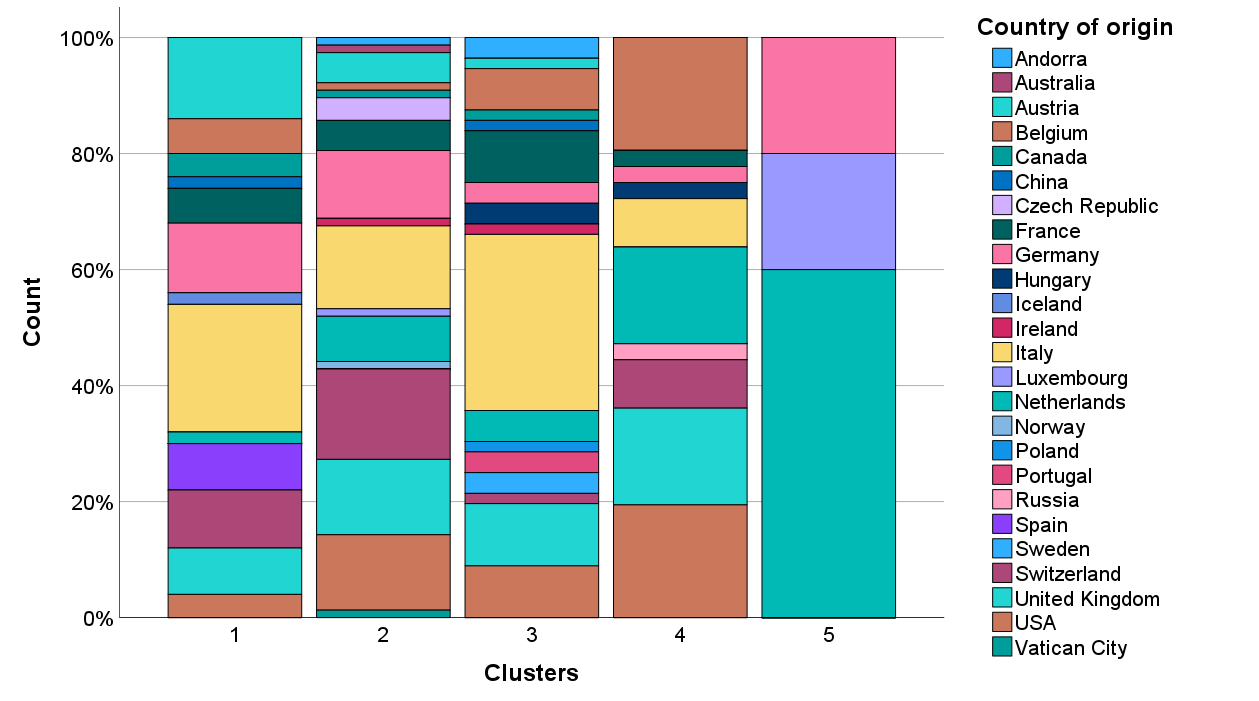** |
| --- | --- |
| **b)** | **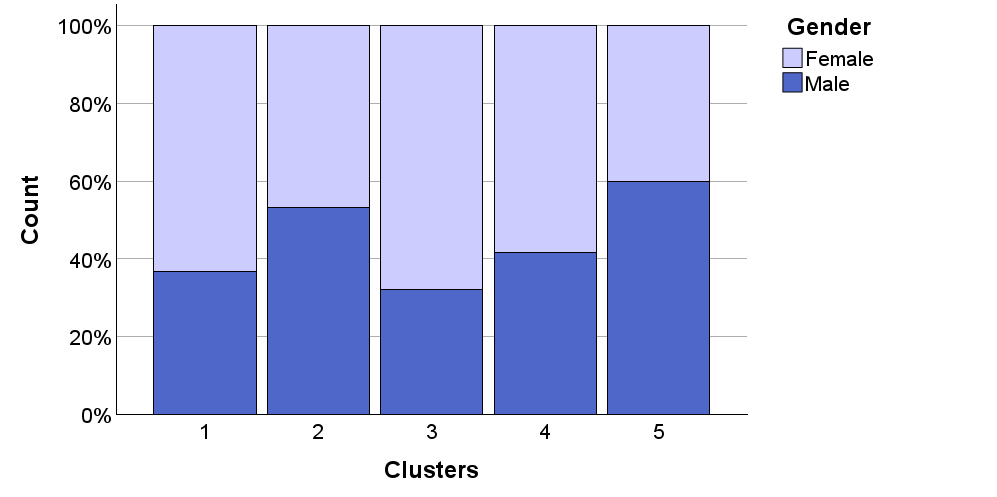** |
| **c)** | **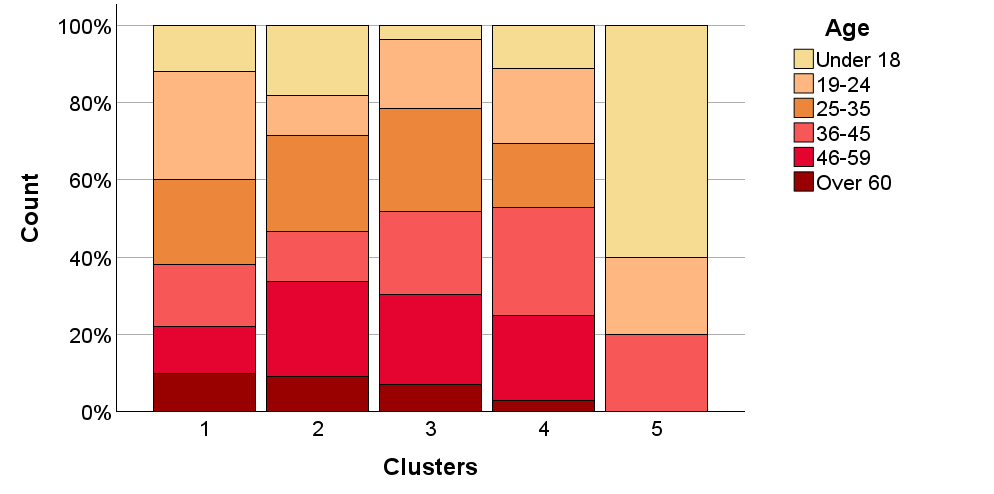** |
| **d)** | **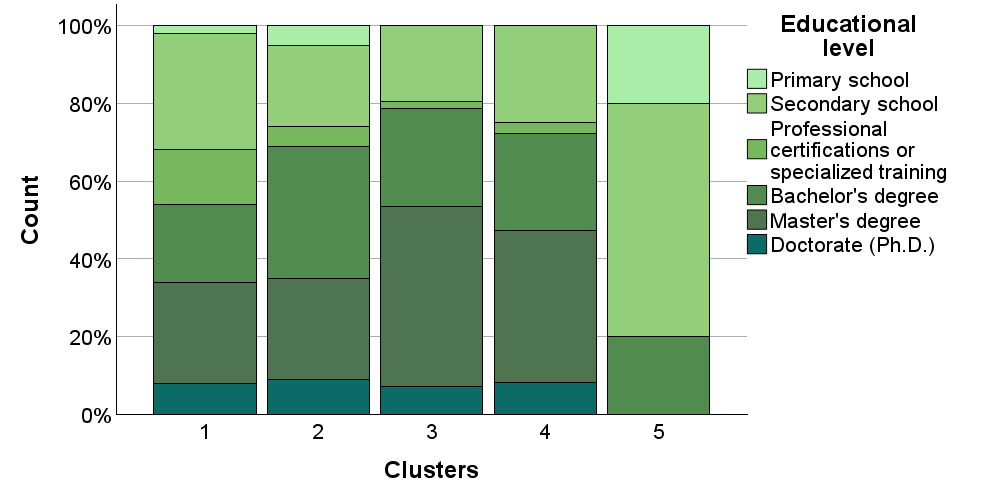** |
| **e)** | ***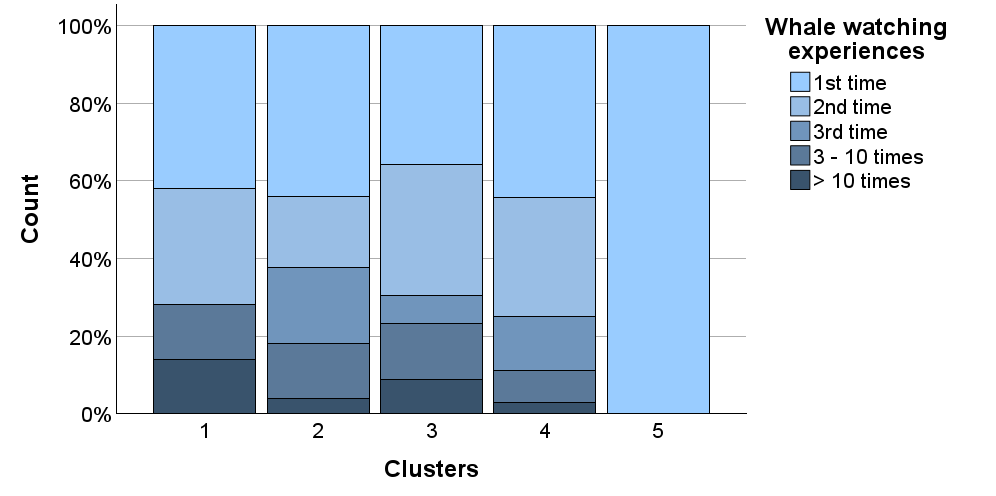*** |

***Figure S5.*** Distribution of respondent clusters in relation to key sociodemographic and experiential variables: a) country of origin; b) gender; c) age; d) educational level; e) whale-watching experiences.

To provide visual context of the fieldwork and citizen science activities, Figure S6 presents a photographic overview from the three study sites. The images illustrate different stages of the process: a) whale-watching tours with participants during cetacean observations, b) the collection of seawater samples directly at sea using sterile buckets, and c) the subsequent eDNA filtering procedures carried out on board with portable vacuum pumps to capture genetic material.

The adopted sampling protocol, including the choice of water volume and filter type, followed recent recommendations for standardizing citizen science-based eDNA collection during whale-watching tours (Rodriguez et al., 2025).

***
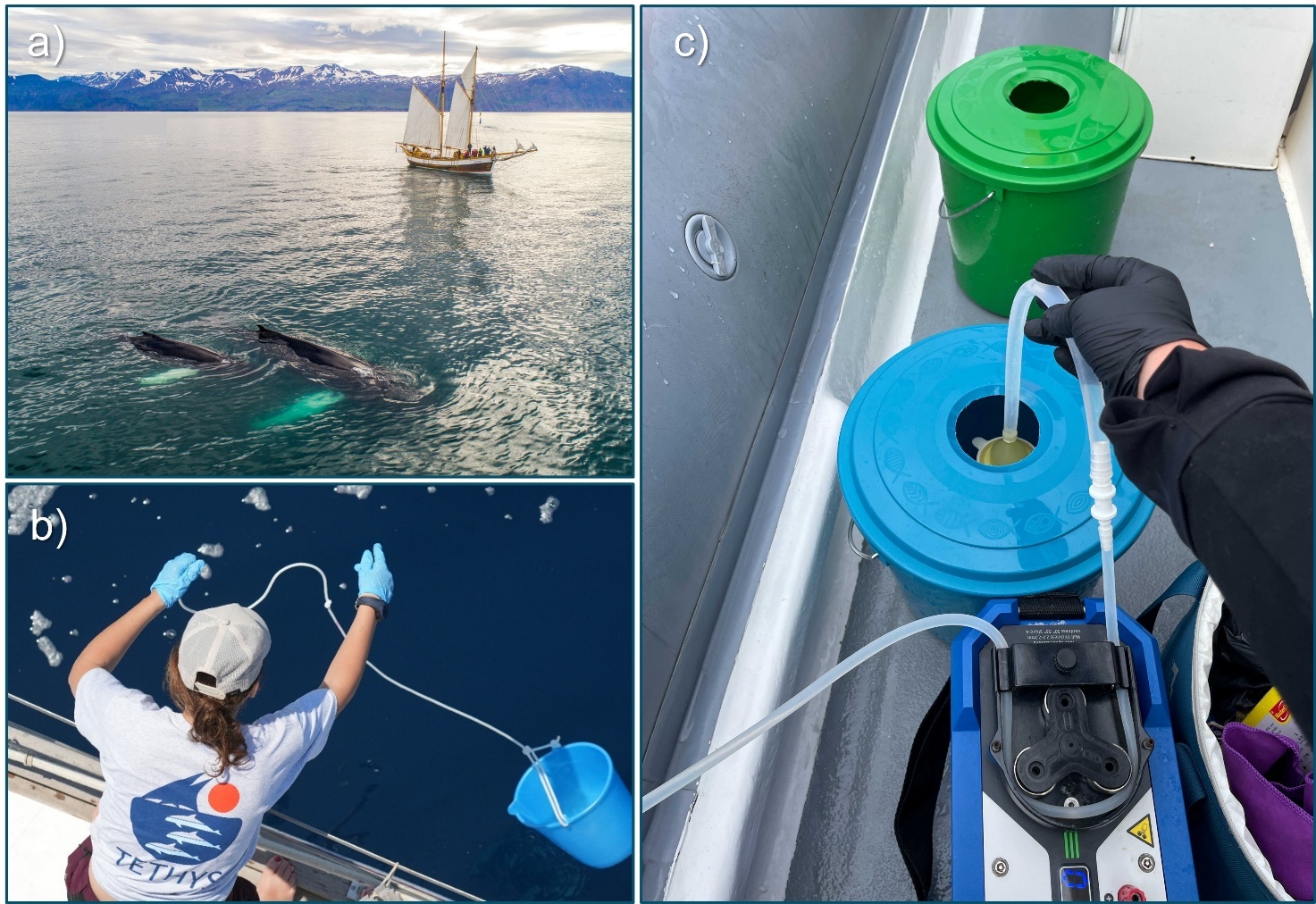
***

***Figure S6.*** *Photographic overview of whale-watching fieldwork and eDNA-informed citizen-science activities. Panels show: a) whale-watching tour in Skjálfandi Bay (Iceland); b) seawater collection at sea using sterile buckets in the Pelagos Sanctuary (Italy); c) onboard eDNA filtration with disposable filter units and a portable vacuum pump in the Azores (Portugal). @Photos by a) North Sailing, b) Tethys Research Institute, and c) CW Azores.*
